# Rare and abundant taxa in *Artemisia desertorum* rhizosphere soils demonstrate disparate responses to drought stress

**DOI:** 10.1007/s44307-025-00070-y

**Published:** 2025-07-03

**Authors:** Mei-Xiang Li, Wen-Hui Lian, Zheng-Han Lian, Xiao-Qing Luo, Ling-Xiang Yue, Jia-Rui Han, Chao-Jian Hu, Shuai Li, Wen-Jun Li, Lei Dong

**Affiliations:** 1 State Key Laboratory of Biocontrol, Guangdong Provincial Key Laboratory of Plant Stress Biology and Southern Marine Science and Engineering Guangdong Laboratory (Zhuhai), School of Life Sciences, Sun Yat‑Sen University, Guangzhou, 510275 PR China; 2 State Key Laboratory of Desert and Oasis Ecology, Key Laboratory of Ecological Safety and Sustainable Development in Arid Lands, Xinjiang Institute of Ecology and Geography, Chinese Academy of Sciences, Urumqi, 830011 PR China

**Keywords:** Desert area, *Artemisia desertorum*, Rhizosphere bacteria, Network complexity, Community assembly process

## Abstract

The growth and adaptability of desert plants depend on their rhizosphere microbes, which consist of a few abundant taxa and numerically dominant rare taxa. However, the differences in diversity, community structure, and functions of abundant and rare taxa in the rhizosphere microbiome of the same plant in different environments remain unclear. This study focuses on the rhizosphere microbial communities of *Artemisia desertorum*, a quintessential desert sand-stabilizing plant, investigating the diversity patterns and assembly processes of rare and abundant taxa across four Chinese deserts: Mu Us, Kubuqi, Tengger, and Ulan Buh. The results show that climatic factors, especially aridity and mean annual precipitation (MAP), significantly influence bacterial community composition and microbial network complexity. The interactions between rare and non-rare taxa are non-random, forming a modular network in which rare taxa serve as central nodes, and their loss could destabilize the network. Rare taxa are primarily shaped by heterogeneous selection, whereas abundant taxa are mainly influenced by dispersal limitation. Functionally, abundant taxa exhibit higher metabolic potential, whereas rare taxa are more involved in processes such as cell motility, indicating distinct ecological roles. These results provide new insights into the ecological functions of rare and abundant taxa in desert rhizosphere communities and highlight the importance of microbial management for desert plant health.

**Supplementary Information:**

The online version contains supplementary material available at 10.1007/s44307-025-00070-y.

## Introduction

Desertification, one of the most severe forms of land degradation (Ren et al. 2022; Zhao et al. 2023), is largely driven by climate change and human activities. In arid environments, resource scarcity poses a major challenge to plant survival (Yang et al. 2023; Zhang et al. 2020). Under such conditions, rhizosphere microorganisms play a crucial role in supporting plant growth and adaptation. There is growing evidence that rhizosphere microorganisms are crucial for plant health, growth, and development, and play an important role in plant fitness under diverse environmental conditions (Lemanceau et al. 2017). Similarly, the rhizosphere microbiome helps desert plants cope with multiple and severe threats such as nutrient deficiency, drought, and salinity (Naeem and Selamoglu 2022﻿, Han et al. 2025). To date, only a limited number of studies have explored the microbial communities associated with desert plants. For example, research has shown that a SynCom of five bacterial strains from the root of the desert plant *Indigofera argentea* can protect tomato plants grown in a non-sterile substrate from high salt stress (Schmitz et al. 2022). Similarly, studies of rhizosphere microorganisms on farms in the Sinai Desert of Egypt have highlighted the significant ecological contributions of microbial diversity in arid regions (Lian et al. 2024). However, the scope and number of such studies remain limited, significantly hindering a systematic understanding of plant–microbe interactions in desert ecosystems and limiting the development of microbe-based strategies for vegetation conservation and ecological restoration.

Microbes, with their vast diversity, are essential in regulating energy flows and nutrient cycles within ecosystems (Muhammad et al. 2025). According to the “seed bank” theory, only a few species dominate the microbial community, while the majority exist in low abundance in the environment (Li et al. 2024; Lynch and Neufeld 2015; Shu and Huang 2020). This division suggests distinct ecological roles: abundant taxa primary drive key ecosystem processes and contribute to community stability (Yang et al. 2022b), whereas rare taxa serve as reservoirs of genetic and functional diversity, profoundly influencing ecosystem resilience and functionality (Liang et al. 2020; Lynch and Neufeld 2015). Environmental factors significantly shape the distribution and ecological strategies of rare and abundant taxa. Rare taxa are more susceptible to environmental selection and typically experience stronger dispersal limitations in ecosystems such as the Tibetan Plateau grasslands, subtropical bays, and oil-contaminated soils (Jiao et al. 2017; Mo et al. 2018; Wan et al. 2021a), In contrast, abundant taxa encounter greater dispersal constraints in saline and agricultural systems (Jiao and Lu 2020b; Wan et al. 2021b). Rising temperatures and alkaline pH conditions further influence microbial assembly by reducing the stochastic processes governing rare taxa while promoting deterministic processes in community assembly (Jiao and Lu 2020b; Mo et al. 2021). These selective pressures highlight the differential responses of rare and abundant taxa to environmental changes, ultimately influencing their functional contributions within the rhizosphere (Lian et al. 2024). Despite their ecological importance, the functional differentiation of rare and abundant taxa in desert plant rhizospheres remains largely unexplored, limiting our understanding of plant–microbe co-evolution and adaptation to extreme environments.

Beyond individual taxonomic groups, microbial interactions within co-occurrence networks play a critical role in shaping community structure and stability (Gao et al. 2022; Ma et al. 2020). Keystone taxa, whether rare or abundant, significantly shape network structure and function through their high connectivity, which is maintained across spatial and temporal scales (Berry and Widder 2014; Hernandez et al. 2021; Kang et al. 2025). For example, climate warming may increase the complexity of microbial networks, thereby affecting ecosystem services and functions (Yuan et al. 2021; Liu et al. 2024). While abundant species are often viewed as key contributors to microbial networks (Yang et al. 2022b), rare species also form strong associations with non-rare (abundant and intermediate) species, thereby contributing to network stability and complexity (Konopka et al. 2015; Nyirabuhoro et al. 2021). However, the impact of climatic factors on the co-occurrence networks of rare and abundant taxa in desert plant rhizospheres remains largely unexplored. This gap underscores the need for further investigation to deepen our understanding of these critical ecological dynamics.

China faces severe desertification, leading to significant ecological and socio-economic consequences. To reconstruct and restore the ecosystem and ecological functions of desertified land, the most effective method adopted by the Chinese government is vegetation restoration (Ala et al. 2014; Liu et al. 2014). *Artemisia desertorum*, commonly known as sand sagebrush, is one of the dominant vegetation types in the desertified areas in northern China, with high tolerance to drought and salinity (Zhang et al. 2020). In this study, we investigated the rhizosphere microbial communities of *A. desertorum* across four severely desertified regions in northern China—the Mu Us, Kubuqi, Tengger, and Ulan Buh deserts (Wang et al. 2017a). These regions are key areas in China’s desertification control efforts and serve as hotspots for *Artemisia desertorum*. This study systematically addresses the following questions: (1) What are the main drivers shaping the distribution patterns of rare and abundant rhizosphere bacterial groups, and do the key predictors differ between them? (2) Are there discernible differences in co-occurrence patterns and community assembly processes between these two groups? (3) What are the potential ecological functional differences between abundant and rare bacterial subcommunities? The results of this study will enhance our understanding of microbial community diversity and assembly processes in the rhizosphere ecosystem of *A. desertorum* and provide a theoretical basis for vegetation restoration in desertified areas and the stabilization of rhizosphere ecosystems.

## Materials and methods

### Soil sampling and experimental design

The geographical distribution of the sampling sites includes the four primary deserts in China and adjacent regions, covering a latitudinal range of 36.23° to 42.22° N and a longitudinal range of 87.72° to 109.78° E. A total of 100 samples were collected from the Tengger Desert (*N* = 24), the Ulan Buh Desert (*N* = 18), the Kubuqi Desert (*N* = 25), and the Mu Us Desert (*N* = 33) (Fig. S1, Table S1). Aridity indices (AI, the ratio of precipitation to potential evapotranspiration) were sourced from the Global Aridity Index and Potential Evapotranspiration Dispersion (GADI) database (Zomer et al. 2022), with AI values above 0.35 classified as natural drylands (Pointing and Belnap 2012). Aridity levels were calculated as 1 − AI (Dong et al. 2024). Site characteristics included a range of elevations from 1024 to 1774 m above sea level. Mean annual temperature (MAT) ranged from 6.4 °C to 8.6 °C, and the mean annual precipitation (MAP) ranged from 102 to 384 mm for the period of 1970 − 2000, with data obtained from https://www.worldclim.org/.

To investigate the composition and diversity of soil biota in a desert ecosystem dominated by *A. desertorum*, soil samples were collected using a natural selection approach (Islam et al. 2023; Zhang et al. 2019). *A. desertorum* is naturally widespread in desert ecosystems, including the study area. Field sampling was conducted in August 2021. Rhizosphere soil was collected from a 10‒20 cm depth near the actively growing roots of *A. desertorum*. The roots were carefully excavated, and the adhering soil was gently separated by tapping the roots to dislodge excess soil. Only soil tightly bound to the roots, forming the rhizosphere, was collected (Li et al. 2023; Lian et al. 2024). After collection, visible plant material was carefully removed, and the soil was sieved through a 2 mm mesh to remove larger debris. The sieved soil was then divided into two portions: one portion was kept in sterile plastic bags and stored at 4 °C for analysis of soil physical and chemical properties, while the other portion was immediately frozen at ‒80 °C for subsequent analysis of soil microbial diversity (Dong et al. 2024; Li et al. 2021).

### Soil properties

All soil samples were analyzed in the laboratory for pH, total nitrogen (TN), nitrate-nitrogen (NO_3_^−^), ammonium-nitrogen (NH_4_^+^), total phosphorus (TP), total organic carbon (TOC), soil water content (SWC), and soil electrical conductivity (SEC). Soil pH was measured using a S210-K pH meter (Mettler Toledo, Germany) in a 1:2.5 (w/v) aqueous solution. TN, NO_3_^−^, and NH_4_^+^ levels were determined using a varioEL C/N analyzer (Westco SmartChem, AMS, Switzerland) in a 1:10 (w/v) aqueous solution. TP was quantified by the molybdenum blue method and TOC was measured via K_2_Cr_2_O_7_ oxidation. SWC was determined gravimetrically by drying the samples at 105 °C for 6 h. SEC, indicates soil salinity, was measured with a HQ14D portable conductivity & TDS meter (Hach Company, Loveland, CO, USA) in a 1:5 (w/v) aqueous solution.

### Molecular and bioinformatics analyses

High‐throughput amplicon sequencing was used to analyze soil bacterial communities (Li et al. 2020). Genomic DNA was extracted from all soil samples using the FastDNA® Spin Kit for Soil (MP Biomedicals, Solon, OH, USA) according to the manufacturer’s protocols. The concentration and purity of the extracted DNA was estimated using NanoDrop^TM^2000 (Thermo Fisher Scientific, Wilmington, DE, USA). The V3-V4 region of the 16S rRNA gene was amplified using the primer pair 338 F (5'-ACTCCTACGGGAGGCAGCA-3') and 806R (5'-GGACTACHVGGGTWTCTAAT-3') for the bacterial community (Dong et al. 2024). PCR amplification conditions were as follows: denaturation at 94 °C for 5 min, denaturation at 94 °C for 30 s, annealing at 52 °C for 30 s, extension at 72 °C for 45 s for 32 cycles, and a final extension at 72 °C for 10 min. Next-generation sequencing library preparation and sequencing were performed on an Illumina NovaSeq 6000 PE250 platform (Illumina, San Diego, USA) at BioMarker Technology Company (Beijing, China).

For each sample, quality-filtered reads were dereplicated with USEARCH 11 (Edgar 2010). After merging paired-end reads, primer sequences were removed using CUTADAPT v2.4 (Martin 2011). Sequences with a maximum expected error greater than 1.0 or a length of < 400 bp for 16S rRNA genes were discarded. The remaining high-quality reads were dereplicated using the unoise3 algorithm (Edgar 2016a), resulting in biological ZOTUs (zero-radius operational taxonomic units). A total of 9,061,482 high-quality sequences were obtained, averaging 53,211 reads per sample. Taxonomy for ZOTUs was assigned using SINTAX (Edgar 2016b) with the SILVA ribosomal RNA database (Release 138) as the reference for bacteria (Quast et al. 2012). ZOTU tables were generated with the “otu_tab” command in USEARCH 11, excluding ZOTUs associated with eukaryotes, chloroplasts, and mitochondria. Sequence counts for downstream analysis were rarefied to the lowest read count across all samples by random resampling.

### Statistical analysis

To reduce random effects on rare subcommunity identification, ZOTUs with fewer than 20 reads were excluded (Jiao and Lu 2020a). ZOTUs with a relative abundance of less than 0.1% across all samples were classified as rare taxa, whereas those with a relative abundance exceeding 1% in one or more samples were designated as abundant taxa (Ji et al. 2020; Liu et al. 2015; Zhang et al. 2019). This classification included both ubiquitously abundant ZOTUs and those occasionally abundant along the transect (Shade et al. 2014). The raw sequences of soil bacteria analyzed in this study are available in the NCBI Sequence Read Archive under BioProject PRJNA1110979.

The *α*-diversity (richness and Shannon diversity index) was calculated for each sample, while *β*-diversity was assessed using Bray–Curtis distance metrics between samples. Non-metric multidimensional scaling (NMDS) with the analysis of similarities (permutations = 999) was used to evaluate variations in soil microbial communities across different deserts. ANOSIM tests were applied to the Bray–Curtis dissimilarity matrix to assess differences in abundant and rare sub-communities among desert ecosystems. Distance-based redundancy analysis (dbRDA) examined the relationships between environmental factors and bacterial communities, including both rare and abundant taxa (Oksanen et al. 2019). Hierarchical partitioning analyses, performed using the “rdacca.hp” R package (Lai et al. 2022), quantified the contributions of each environmental variable to the variations in bacterial communities. The Mantel test, using the “vegan” R package, assessed the correlation between the microbial community matrix and the soil properties matrix. Different distance algorithms were applied, with soil physical and chemical properties based on Euclidean distance and microbial community data based on Bray–Curtis distance.

To investigate community stability and interspecific relationships, we conducted a co-occurrence network analysis based on SparCC correlations. Only ZOTUs detected in more than one-third of the samples from each reactor were included to reduce low-frequency noise. SparCC correlations were calculated using FastSpar (version 1.0.0) (Watts et al. 2019). First, we generated an initial correlation matrix using FastSpar and created 999 bootstrap samples to assess correlation stability. These samples were analyzed in parallel to produce correlation and covariance matrices. Then, we calculated *P*-values for each correlation with 999 permutations, applying a final filter to retain only correlations with *r* > 0.45 and *P-*value ≤ 0.05 before constructing the networks. A biological network at the ZOTU level was constructed using the R package “igraph” and visualized with Gephi (v0.9.2) (Jiang et al. 2017a; 2017b). To assess network structure, 9999 Erdös-Rényi random networks with the same number of nodes and edges as the real networks were generated using the ‘igraph’ package in R v4.0.3. (Csardi et al. 2006; Tian et al. 2022). Key topological characteristics, including average clustering coefficient, modularity, and average shortest path length, were compared between the real and random networks (Mo et al. 2021). Node connectivity was classified using within-module connectivity (Zi) and among-module connectivity (Pi) to identify keystone taxa (Guimera and Amaral 2005). This analysis followed previously established methodologies (Deng et al. 2012; 2016; Olesen et al. 2007; Shi et al. 2016; Zhou et al. 2010). Subnetworks were created from the comprehensive bacterial network by retaining taxa associated with each site using the subgraph() function in the igraph package, as described by Ma et al. (2016).

A null-model approach, based on the beta-nearest taxon index (*β*NTI) and the Raup-Crick metric (RC_bray_), was employed to investigate the contributions of ecological processes to microbial community assembly and differences in ecological patterns (Dong et al. 2024; Hou et al. 2022; Zhou and Ning 2017; Zhang et al. 2022). *β*NTI was calculated to quantify the deviation between the observed *β*MNTD distribution and the expected *β*MNTD. A |*β*NTI|> 2 indicates that community assembly is primarily dominated by deterministic processes (*β*NTI < ‒2 indicates homogeneous selection, while *β*NTI > 2 indicates heterogeneous selection). In contrast, a |*β*NTI|< 2 indicates that community assembly is dominated by stochastic processes. The Raup-Crick (RC_Bray_) was combined with *β*NTI to further partition pairwise comparisons assigned to stochastic processes (|*β*NTI|< 2). Specifically, |*β*NTI|< 2 and |RC_Bray_|> 0.95 indicate that community assembly is driven by probabilistic dispersal (RC_Bray_ > + 0.95 indicates dispersal limitation, while RC_Bray_ < −0.95 indicates homogenizing dispersal). Finally, |*β*NTI|< 2 and |RC_Bray_|< 0.95 suggest that community assembly is dominated by undominated processes (Stegen et al. 2013).

The potential metabolic pathways of rare and abundant taxa from the rhizosphere of *A. desertorum* were predicted using PICRUSt2. The differences between rare and abundant taxa were analyzed using Statistical Analysis of Metagenomic Profiles (STAMP) (Wang et al. 2020). The resulting functional predictions were then organized according to the Kyoto Encyclopedia of Genes and Genomes (KEGG) pathways.

In the statistical analysis, we applied the Benjamini-Hochberg (BH) method to adjust *P*-values for multiple comparisons and calculated Cohen’s d to measure effect sizes for significant group differences. All of the analyses were conducted in R version 4.3.2.

## Results

### Soil physicochemical properties

Samples from different desert ecosystems, including Mu Us, Kubuqi, Tengger, and Ulan Buh deserts, were analyzed for various physicochemical properties as shown in Fig. S2. Significant differences were observed in soil properties, especially in MAP and SWC, which showed a pronounced decreasing trend in the four deserts. In contrast, Aridity and MAT exhibited a significant increasing trend. The Ulan Buh Desert had a significantly higher pH than the Mu Us (cohen’s d = 0.575, *P* < 0.05), Kubuqi (cohen’s d = 0.965, *P* < 0.05), and Tengger (cohen’s d = 0.887, *P* < 0.05) deserts, all of which were alkaline. No significant differences in SEC were observed among the desert ecosystems. Additionally, NO_3_^−^ content was significantly lower in the Tengger Desert compared to the other desert ecosystems.

### Diversity and compositions of rare and abundant taxa

**Fig. 1:**
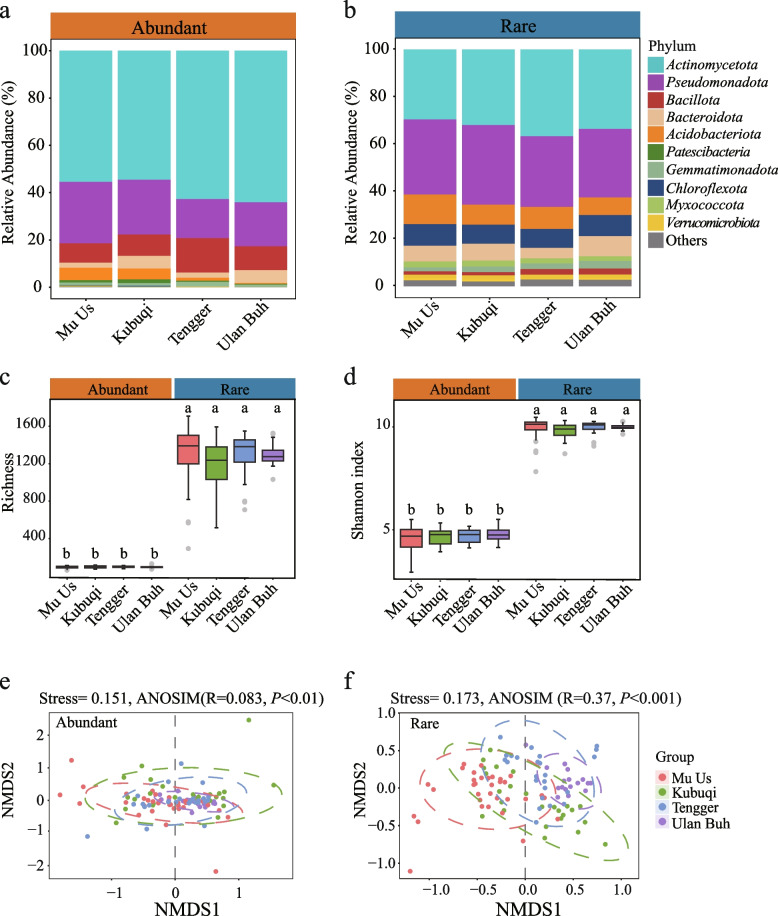
Composition and diversity of abundant and rare taxa in the rhizosphere of *Artemisia desertorum*. **a**, **b** Bacterial community composition in the rhizosphere of *A. desertorum* across different desert ecosystems. **c**, **d** Analysis of alpha-diversity (richness and Shannon diversity index) of bacterial communities. **e**, **f** Non-metric multidimensional scaling (NMDS) ordination of abundant and rare subcommunities. 95% confidence ellipses were shown around the samples grouped based on different desert systems. Group differences were tested based on ANOSIM analysis. Lowercase letters denote the differences among different deserts at *P* < 0.05

A total of 9,796 ZOTUs were identified across all samples, comprising 1,068,658 sequences. Rare bacteria represented 58.25% of the total bacterial richness (5,706 ZOTUs), but their total relative abundance accounted for only 20.26% of the entire community. In contrast, abundant bacteria comprised just 2% of the total richness (217 ZOTUs), but their relative abundance contributed to 24.65% (Table S2). Abundant bacterial ZOTUs were associated with 11 bacterial phyla, whereas the rare subcommunity consisted of 30 phyla. At the phylum level, the abundant subcommunities were dominated by *Actinomycetota* (58.58%), *Pseudomonadota* (22.14%) and *Bacillota* (10.19%) (Fig. 1a, Table S3). In contrast, the rare bacterial subcommunities were mainly composed of *Actinomycetota* (32.7%), *Pseudomonadota* (30.99%), *Acidobacteriota* (10.1%), *Chloroflexota* (8.61%) and *Bacteroidota* (6.61%) (Fig. 1b, Table S3). The relative abundances of these phyla varied across different desert ecosystems for both the abundant and rare subcommunities (Fig. S3). At the family level, the abundant subcommunities were mainly composed of *Micrococcaceae*, *Nocardioidaceae*, and *Bacillaceae*, whereas the rare subcommunities were predominantly composed of unknown taxa (Fig. S4). Across different soil samples, the abundant subcommunities were observed to be more widespread than the rare ones. Specifically, 195 out of 217 abundant ZOTUs (89.86%) were found in more than 50% of the samples, whereas only 145 rare ZOTUs (2.54%) were present in more than 50% of the samples. Generally, the *α*-diversity of rare subcommunities, as measured by the ZOTU Shannon diversity index and richness, was significantly higher than that of abundant subcommunities in the rhizosphere of *A. desertorum*. The richness of rare subcommunities was 703.1% to 1338.4% higher than that of abundant ones (Fig. 1c), and the Shannon diversity index was 76.2% to 164.75% greater than that of abundant taxa (Fig. 1d). Furthermore, there were no significant differences in the Shannon diversity index and richness among the four deserts (Fig. 1c, d), indicating a relatively stable bacterial community composition in *A. desertorum* rhizosphere soils. However, when bacterial diversity (*β*-diversity) between samples was assessed using the Bray–Curtis dissimilarity index, rare subcommunities exhibited significantly higher dissimilarity compared to abundant subcommunities (all *P*_adj_ < 0.001) (Fig. S5). NMDS analysis revealed distinct clustering of rhizosphere bacteria from the Mu Us, Kubuqi, Tengger, and Ulan Buh deserts in the ordination space of both abundant subcommunities (NMDS, stress = 0.151; ANOSIM R = 0.083, *P* < 0.001) (Fig. 1e) and rare subcommunities (NMDS, stress = 0.173; ANOSIM R = 0.37, *P* < 0.001) (Fig. 1f). To test the robustness of our results, we reanalyzed diversity and community composition using an alternative abundance threshold. ZOTUs with fewer than 20 reads were excluded to minimize random effects. Following Jiao and Lu (2020b) and Zhang et al. (2021b), ZOTUs were defined “abundant” as those having relative abundances above 0.1% of the total sequences, and “rare” as those having relative abundances below 0.01%. This classification identified 87 abundant and 7,850 rare ZOTUs. The resulting diversity patterns and community structures were highly consistent with our main findings, confirming the reliability of the results (Fig. S6, Table S4).

**Fig. 2:**
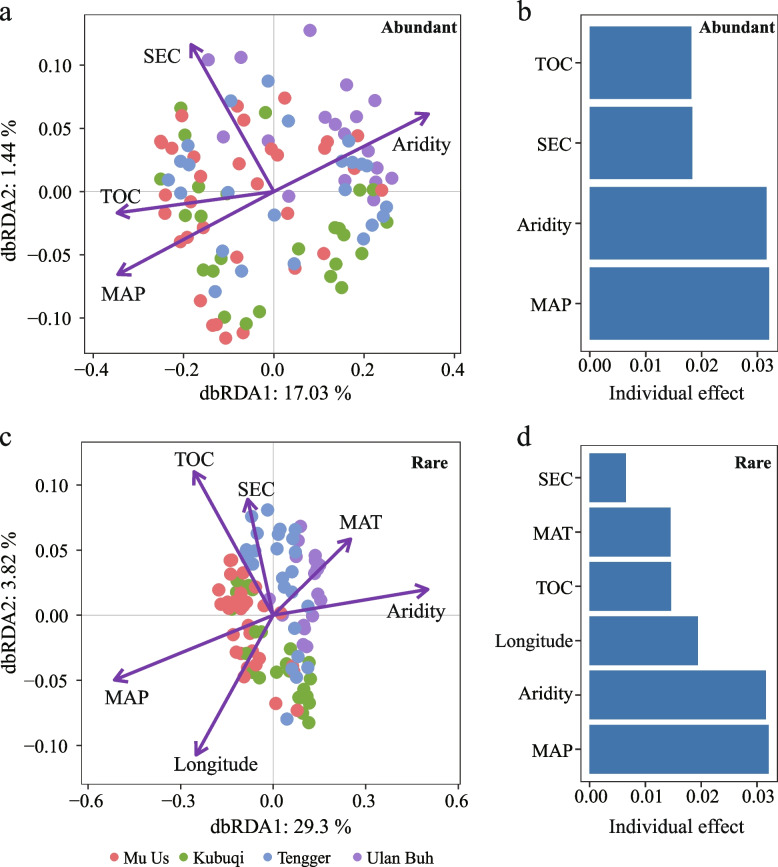
Effects of environmental factors on abundant and rare bacterial subcommunities. **a**, **c** Distance-based redundancy analysis (dbRDA) showing the relationship between the structure of rare and abundant taxa and environmental factors. **b**, **d** Hierarchical partitioning of each environmental factor explaining the structure of rare and abundant subcommunities (*P* < 0.05). Full abbreviations: MAP, mean annual precipitation; MAT, mean annual temperature; SEC, soil electrical conductivity; TOC, total organic carbon

According to the dbRDA analysis, the main factors influencing the composition of the abundant subcommunities were MAP, aridity, TOC, and SEC (Fig. 2a). Hierarchical partitioning analyses indicated that MAP and aridity were the most important factors shaping the *A. desertorum* rhizosphere community (Fig. 2b). However, the rare subcommunity was influenced by a broader range of environmental factors, including longitude, MAP, aridity, MAT, TOC, and SEC (Fig. 2c). Hierarchical partitioning analysis further indicated that MAP and aridity were also the major contributing factors (Fig. 2d). Further Mantel test analysis showed that the composition of abundant subcommunities was primarily influenced by aridity and MAP (all *P*_*adj*_ < 0.05), whereas the composition of rare subcommunities was primarily influenced by MAP, aridity, MAT, longitude and latitude (all *P*_*adj*_ < 0.01), and secondarily by elevation (*P*_*adj*_ < 0.05) (Table S5). Interestingly, MAP and aridity emerged as the dominant influencing factors for both rare and abundant subcommunities (Fig. S7, Table S5). We also examined the relationship between environmental factors and the top ten bacterial taxa. Both rare and abundant taxa were strongly influenced by aridity and MAP (Fig. S8).

### Microbial interaction networks

**Fig. 3:**
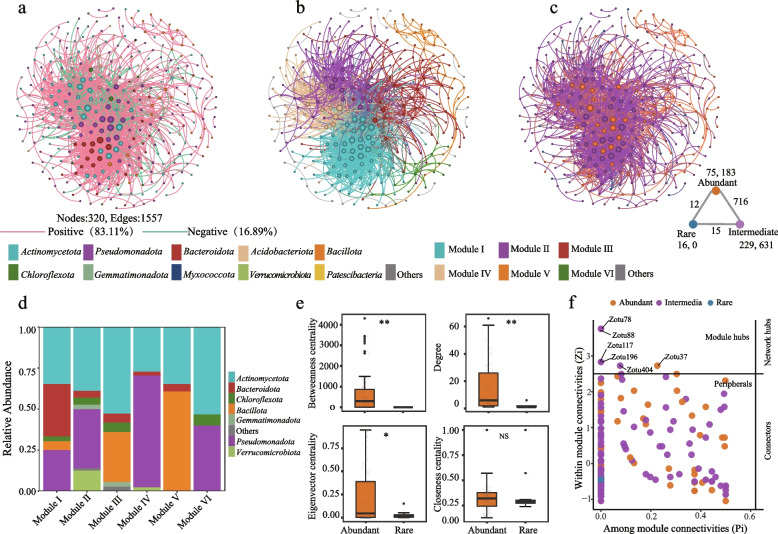
Co-occurrence network of *A. desertorum* rhizosphere systems. **a** Co-occurrence network colored for phylum. **b** Module structure of the co-occurrence network. **c** Network properties colored by abundant or rare species, with external and internal connections among each subcommunity shown in the bottom right. **d** Composition of each module at phylum level. **e** Comparison of node-level topological characteristics between abundant and rare taxa. **f** Distribution of keystone nodes. NS, *P* > 0.05, **P < 0.01, *P ≤ 0.05 (Wilcoxon test, Benjamini Hochberg·correction)

In our investigation of symbiotic patterns among bacterial communities in the rhizosphere of *A. desertorum*, we constructed a co-occurrence network consisting of 320 nodes and 1557 edges (Fig. 3a). The empirical network exhibited higher modularity, average clustering coefficient, and average path length than the Erdös-Rényi random networks, indicating “small-world” properties and a modular structure (Table S6). The entire network was primarily organized into six modules with distinct associations (Fig. 3b). Rare taxa interact more frequently with non-rare taxa (abundant and intermediate) than with conspecifics (Fig. 3c). Module I was dominated by taxa from *Actinomycetota*, *Bacteroidota*, and *Pseudomonadota*; Module II primarily consisted of *Pseudomonadota* and *Actinomycetota*; Module III mainly comprised *Actinomycetota*, and *Bacillota*; Module IV consisted mainly of *Pseudomonadota* and *Actinomycetota*; Module V predominantly included *Actinomycetota*, and *Bacillota*; Module VI was primarily composed of *Actinomycetota* (Fig. 3d). We analyzed the topological characteristics of nodes in both rare and abundant subcommunities, including degree, closeness centrality, betweenness centrality, and eigenvector centrality. Abundant taxa had significantly higher eigenvector centrality (cohen’s d = 0.866, *P* < 0.05), betweenness centrality (cohen’s d = 0.949, *P* < 0.05), and degree (cohen’s d = 1.308, *P* < 0.05) compared with rare taxa (Fig. 3e). These results suggest that variations in network complexity may alter the roles of individual network members. We identified six module hubs within the co-occurrence network, based on their Zi and Pi scores. These nodes were crucial as keystone nodes that significantly influence the network structure (Fig. 3f). The identified keystone species included *Micrococcaceae*, *Xanthobacteraceae*, *Sphingomonadaceae*, *Nitrospiraceae*, *Oxalobacteraceae*, and a potentially novel genus within *Chloroflexota*, all of which belonged to the abundant or intermediate subcommunities (Table S7).

**Fig. 4:**
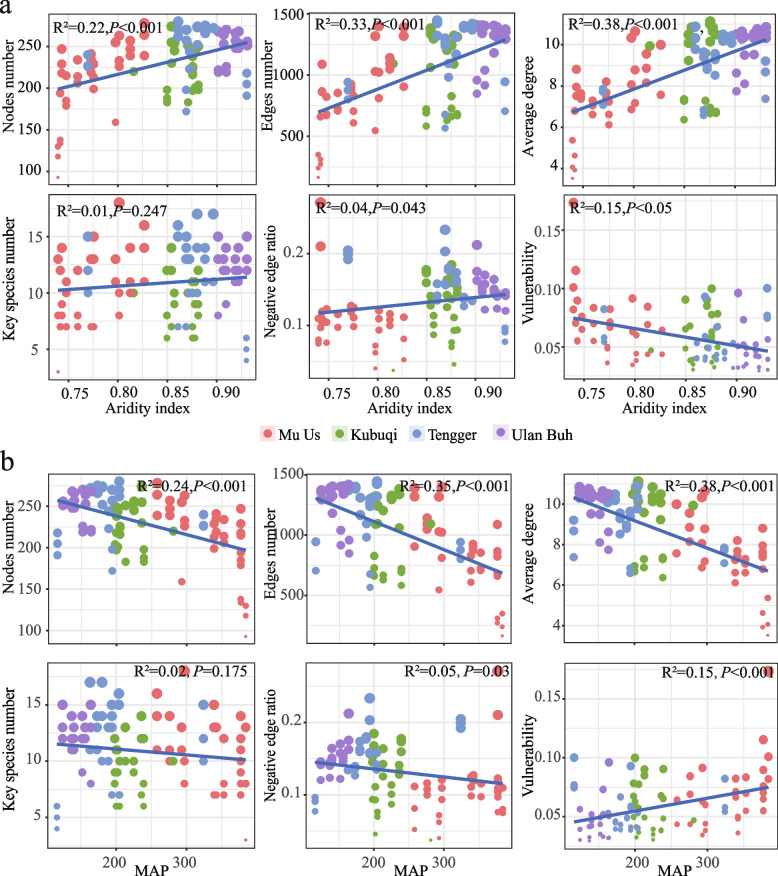
Linear regression models of subnetwork topological structure and climatic factors. **a** Linear regression analysis between subnetwork topology and aridity. **b** Linear regression analysis between subnetwork topology and mean annual precipitation (MAP)

To investigate how climatic factors, such as aridity and mean annual precipitation (MAP), influence the complexity of microbial networks, sub-networks of rhizospheric bacteria from *A. desertorum* were created using samples collected across four deserts. The topological structures of these “sub-networks” were computed for each sample, and regression analyses were performed to examine the relationships between various network topological parameters and aridity or MAP. The results revealed that with increasing aridity, there were significant increases in the total number of nodes (*R*^2^ = 0.22, *P* < 0.001), edges (*R*^2^ = 0.33, *P* < 0.001), and average degree (*R*^2^ = 0.38, *P* < 0.001). In addition, the proportion of negative correlations also increased significantly (*R*^2^ = 0.04, *P* < 0.05), while network vulnerability decreased significantly (*R*^2^ = 0.15, *P* < 0.001). However, the number of keystone nodes showed no significant change (Fig. 4a). In contrast, increasing MAP exhibited an opposite trend (Fig. 4b).

### Community assembly in different subcommunities

**Fig. 5:**
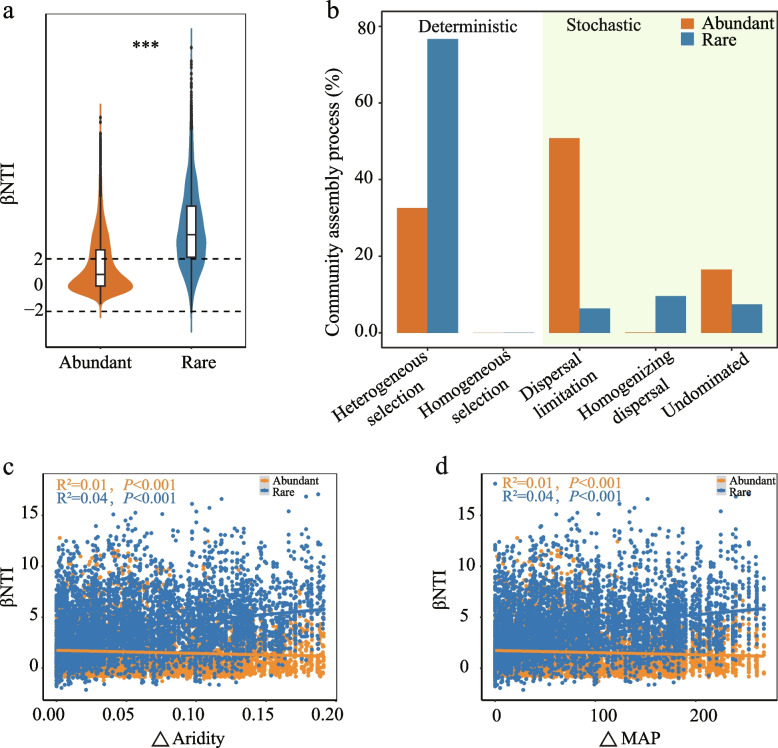
Mechanism of bacterial community assembly in *A. desertorum* rhizosphere. **a** Distribution of *β*NTI values for abundant and rare taxa. **b** The relatively explained ecological processes of abundant and rare subcommunities based on the values of *β*NTI and RC_bray_. The relationships between bacterial phylogenetic turnover (*β*NTI) and **c** aridity and **d** mean annual precipitation (MAP). ***P < 0.001. Linear regressions models (blue lines for rare subcommunities and orange lines for abundant subcommunities) and associated correlation coefficients are provided on each panel

Null-model approaches were employed to investigate the assembly processes of both rare and abundant taxa, using *β*NTI and RC_bray_ values. The findings indicated that the assembly of abundant taxa was primarily driven by stochastic processes (67.43%), while deterministic processes dominated the assembly of rare taxa (76.7%). Within deterministic processes, heterogeneous selection had a stronger influence on rare taxa (76.64%) than on abundant taxa (32.57%). In contrast, the assembly of abundant taxa was less phylogenetically structured and primarily driven by dispersal limitation (50.81%) (Fig. 5a, b). To further elucidate turnover patterns across different desert ecosystems, turnover rates were calculated individually for the four deserts. For abundant taxa, the role of stochastic clustering processes in the aggregation of the Kubuqi Desert community (49%) was smaller than in the Mu Us (73.49%), Tengger (88.77%), and Ulan Buh (71.35%) deserts. Deterministic processes had the greatest impact on the assembly of rare subcommunities in Mu Us Desert (80.68%) and the least impact on Ulan Buh Desert (32.68%) (Fig. S9). With increasing differences in MAP and aridity, the *β*NTI of rare subcommunities increased significantly (*P* < 0.001) (Fig. 5c), while that of abundant subcommunities showed a significant decreasing trend (*P* < 0.001) (Fig. 5d). These findings reveal that greater differences in MAP and aridity shifted the assembly of abundant taxa from stochastic processes to heterogeneous selection, whereas rare taxa exhibited the opposite pattern.

### Bacterial potential functions of different subcommunities

**Fig. 6:**
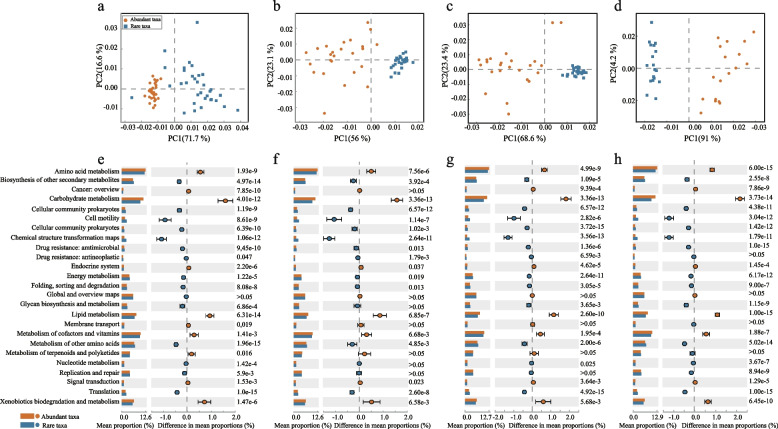
Functional differences between abundant and rare bacterial subcommunities in the *A. desertorum* rhizosphere ecosystem. **a**–**d** Principal component analysis (PCA) shows the distinct metabolic pathways between rare and abundant taxa ground on KEGG Database level 1. **e**–**h** Comparison of the relative abundance of the top 25 functional profiles between abundant and rare taxa based on KEGG Database level 2 using statistical analysis of metagenomic profiles (STAMP). **a**, **e** Mu Us Desert, **b**, **f** Kubuqi Desert, **c**, **g** Tengger Desert, **d**, **h** Ulan Buh Desert

Based on PICRUSt 2, we predicted the potential functions of rare and abundant subcommunities, revealing that metabolism (KEGG level 1) was the dominant category among the KEGG pathways (Fig. S10). Approximately 74.8% to 77.6% of the sequences were associated with metabolism, 8.6% to 9.4% in genetic information processing, 2.2% to 2.4% in environmental information processing, 4.3% to 6.2% in cellular processes, 2.4% to 2.6% in organismal systems, and 4.2% to 4.9% in human diseases. PCA visualization of the predicted metabolic pathways at KEGG level 1 (Fig. 6a-d) revealed significant differences in metabolic functions between rare and abundant subcommunities in the rhizosphere of *A. desertorum*. STAMP analysis revealed significant differences in the metabolic potential at KEGG level 2 between rare and abundant taxa. Among the top 25 functional groups at level 2, carbohydrate metabolism, amino acid metabolism, and metabolism of cofactors and vitamins were the most prevalent pathways. Abundant subcommunities exhibited significantly higher relative abundances in these pathways compared to rare subcommunities. Additionally, abundant taxa displayed significantly higher metabolic potential in xenobiotic biodegradation and metabolism, lipid metabolism (*P* < 0.05), possibly due to their elevated metabolic activity, which enhances their capability to metabolize exogenous substances. Conversely, rare taxa demonstrated higher potential in cell motility and energy metabolism, suggesting their fundamental role in promoting rhizosphere formation and maturation. The functional profiles of rhizospheric bacteria showed consistent trends across the four deserts. Among the top 25 functional pathways, significant differences between rare and abundant subcommunities were observed in 24 pathways in the Mu Us Desert, 18 in the Kubuqi Desert, and 21 in both the Tengger and Ulan Buh Deserts (Fig. 6e-h). However, since PICRUSt2 analysis provides only a broad prediction of bacterial community functions, further metagenomic analysis is necessary for a more accurate assessment of the functional distribution in rhizospheric bacteria.

## Discussion

The study used high-throughput sequencing technology to examine the microbial community in the rhizosphere of *A. desertorum*. The abundant subcommunity included 11 microbial phyla, while the rare subcommunity included 30 microbial phyla (Table S3). Dominant phyla, such as *Actinomycetota*, *Pseudomonadota*, *Bacillota*, and *Bacteroidota*, are well-documented in desert plant rhizospheres, though their relative abundances vary (Lian et al. 2024; Yang et al. 2023; Zhu et al. 2024) (Fig. 1a, b; Fig. S3). Phyla with a large number of generalists likely play an important role in maintaining the rhizosphere ecosystem of *A. desertorum*. For instance, most *Pseudomonadota* are copiotrophic bacteria, with *α*-, *β*-, and *γ*- *Pseudomonadota* typically linked to increased organic carbon inputs (Blaya et al. 2013). However, in arid and nutrient-limited environments, *Pseudomonadota* may be even more crucial, as some members are involved in bacteriochlorophyll-dependent photosynthesis (Boldareva-Nuianzina et al. 2013; Raymond 2008).

Environmental heterogeneity and ecological processes shape distinct distribution patterns of abundant and rare taxa across different ecosystems, including grasslands, farmlands, and wetlands (Jiao and Lu 2020b; Ji et al. 2020; Wan et al. 2021a). In *A. desertorum* rhizosphere ecosystems, rare and abundant subcommunities exhibit notable differences. The *α*-diversity of rare subcommunities is substantially higher than that of abundant ones (Fig. 1c, d), consistent with previous studies (Jiao et al. 2017; Xiong and Wan 2023). This increased diversity likely results from frequent species turnover within the rare biosphere, contributing to ecosystem stability and functional redundancy (Cordero et al. 2016; Jiao et al. 2020c; Lynch and Neufeld 2015; Shade et al. 2014). In contrast, *β*-diversity shows significant spatial variation across desert ecosystems, suggesting that habitat-driven selection plays a greater role in structuring community composition than in shaping local species richness. This aligns with reports from grasslands and agricultural systems, where microbial *β*-diversity varies due to differences in environmental filtering, dispersal limitation, and biotic interactions (Jiao et al. 2017; Ren et al. 2018).

By revealing the co-occurrence patterns of abundant and rare taxa, this study provides insights into the ecological interactions that shape the rhizosphere microbiome. The network structure shows non-random connectivity and high modularity, similar to patterns observed in soil and aquatic systems (Chen et al. 2020a, b; Egidi et al. 2019; Wang et al. 2017b). In particular, the limited covariation between rare and abundant taxa (Fig. 3c) suggests distinct ecological strategies, possibly driven by differential responses to resource availability and environmental selection. Network topological properties, such as degree, closeness, and betweenness centrality, show that abundant taxa occupy central positions, enabling greater information flow within the network (Fig. 3e). Six module hubs were identified, all belong to the abundant and intermediate subcommunities (Table S7). These module hubs are key in structuring the microbial co-occurrence network. As highly connected taxa within modules, they act as ‘ecosystem engineers’, promoting local cohesion and intra-module interactions. Their presence helps maintain the network’s structural integrity and functional robustness (Banerjee et al. 2018; Hu et al. 2017; Shi et al. 2020). Our results indicate that rare taxa mainly interact with non-rare taxa rather than forming tightly connected clusters, which suggests they may act as ecological stabilizers. This aligns with findings that rare taxa enhance community resilience by buffering against environmental fluctuations (Konopka et al. 2015).

Our assessment of microbial community assembly reveals that environmental factors not only significantly influence the overall microbial community but also have distinct effects on rare and abundant taxa. Null model analysis suggests that deterministic processes, particularly heterogeneous selection, dominate rare taxa assembly, while stochastic processes, especially dispersal limitation, dominate abundant taxa (Fig. 5b). This supports previous studies reporting that abundant taxa are more limited by dispersion than rare taxa in China’s Loess Plateau (Shu et al. 2021) and in agricultural ecosystems in eastern China (Jiao and Lu 2020a). Dispersal limitation affects abundant taxa significantly (Fig. 5b), likely due to their wider distribution, higher dispersal potential, and involvement in more frequent dispersal events (Liu et al. 2015; Jiao et al. 2018; Yang et al. 2022a). The high heterogeneous selection of rare taxa could be partially explained by their lower relative abundance in desert ecosystems, as microorganisms with relatively low abundance are more susceptible to environmental filtering (Jiao and Lu 2020a; Zhu et al. 2023). Abiotic factors regulate the balance between stochastic and deterministic processes in soil microbial communities. For instance, salinity plays a significant role in this balance for both rare and abundant taxa in saline agricultural soils (Wan et al. 2021b), while soil moisture content is crucial for taxonomic group assembly in coastal wetlands (Gao et al. 2020). This study highlights the significant role of mean annual precipitation (MAP) and aridity in microbial assembly, aligning with findings from other arid ecosystems (Maestre et al. 2015, 2021; Xiong et al. 2020; Zhao et al. 2020). In arid regions, precipitation plays a crucial role in determining the spatial distribution of microbes (Tripathi et al. 2017). Precipitation directly increases soil moisture, facilitating microbial survival, while indirectly altering soil pH and nutrient availability, which shape microbial composition (Griffiths et al. 2011).

Rare and abundant taxa in the rhizosphere of *A. desertorum* play distinct functional roles, reflecting their adaptive strategies in arid conditions. The microbial functional potential spans several pathways (Fig. 6e-h), with metabolic functions dominating, followed by environmental information processing, cellular processes, and genetic information processing (Fig. S10). Amino acid metabolism is the most enriched pathway, likely due to desert conditions that accelerate amino acid turnover and limit biosynthetic capacity (Massalha et al. 2017). This metabolism supports microbial survival, reproduction, and stress resistance in arid conditions (Rahman et al. 2015). Functional differences between rare and abundant taxa highlight their distinct ecological roles. Abundant taxa show higher metabolic activity, regulating plant immune responses, modulating growth, and adapting to environmental stress (He et al. 2024; Henning et al. 2016; Yu et al. 2021; Zhang et al. 2021a. Their enriched carbohydrate and lipid metabolism pathways allow them to optimize resource use and support competing species (Tang and Zhou 2011). In contrast, rare taxa emphasize rapid environmental responses, such as enhanced cell motility, energy metabolism, and replication and repair, which align with their dormant state during unfavorable conditions and activation under favorable conditions (Campbell et al. 2011; Zhu et al. 2022, 2023). Environmental disturbances, including drought and precipitation, play a critical role in shaping these functional differences (Malik et al. 2020; Wan et al. 2023). The enrichment of amino acid metabolism under arid conditions reflects adaptive strategies to cope with water limitation and nutrient stress, while the metabolic flexibility of abundant taxa enhances resilience to environmental fluctuations (Chen et al. 2021; Zhang et al. 2023). Rare taxa, with their replication and repair potential, act as an ecological ‘seed bank,’ stabilizing microbial communities during disturbances (Lynch and Neufeld 2015; Wisnoski and Lennon 2021; Jia et al. 2018). These findings highlight the functional differentiation between rare and abundant taxa and how environmental disturbances modulate their ecological roles (Coleine et al. 2024; Pan et al. 2022).

It is important to note that the functional predictions here are based on PICRUSt2, which infers potential metabolic functions from 16S rRNA gene data using reference genomes and phylogenetic assumptions. This approach may not fully capture the functional diversity of uncharacterized or low-abundance taxa, nor does it reflect gene expression or activity. Future studies integrating metagenomic or metatranscriptomic approaches are needed to validate these predictions and provide a more accurate view of microbial functional capacities in desert rhizospheres.

## Conclusions

This study provides the first comprehensive analysis of rare and abundant soil microbial communities in the rhizosphere of *Artemisia desertorum* across four major deserts in northern China. The results show that while bacterial diversity in the rhizosphere of *A. desertorum* lacks a clear spatial distribution pattern, rare taxa exhibit greater diversity and distinct spatial distribution patterns, primarily influenced by climatic factors such as mean annual precipitation (MAP) and aridity. Co-occurrence network analysis highlights frequent interactions between rare and non-rare taxa, with abundant species occupying more central positions. Climatic factors significantly influence community stability, and bacterial community assembly is shaped by both deterministic and stochastic processes. Specifically, heterogeneous selection drives the assembly of rare subcommunities, while stochasticity plays a stronger role in abundant taxa. Functional analyses suggest that abundant taxa contribute more to plant-associated metabolic activities, whereas rare taxa may play crucial roles under changing environmental conditions. These findings enhance our understanding of microbial community assembly in arid ecosystems and emphasize the ecological significance of rare microbes. To support desert ecosystem management, future research should explore strategies for harnessing beneficial microbial functions, particularly by promoting rare microbial diversity, to enhance soil health and improve plant resilience in arid environments.

## Supplementary Information

Supplementary Material 1

## Data Availability

The data that support the findings of this study are openly available in NCBI SRA database at https://www.ncbi.nlm.nih.gov, reference number PRJNA1110979.
