# Supplementary material for: Rare and abundant taxa in Artemisia desertorum rhizosphere soils demonstrate disparate responses to drought stress
Source: Adv Biotechnol (Singap). 2025 Jul 3;3(3):21. doi: 10.1007/s44307-025-00070-y (PMC12229302; doi:10.1007/s44307-025-00070-y)
Supplement: Supplementary file 1 — Supplementary Material 1 [file 44307_2025_70_MOESM1_ESM.docx]

## **Supplementary materials**

**Rare and Abundant Taxa in *Artemisia desertorum* Rhizosphere Soils Demonstrate Disparate Responses to Drought Stress**

**Mei-Xiang Li ^a^, Wen-Hui Lian ^a^, Zheng-Han Lian ^a^, Xiao-Qing Luo ^a^, Ling-Xiang Yue ^a^, Jia-Rui Han ^a^, Chao-Jian Hu ^a^, Shuai Li ^a,b^, Wen-Jun Li ^a,b^*, Lei Dong ^a,^***

**^a^ State Key Laboratory of Biocontrol, Guangdong Provincial Key Laboratory of Plant Stress Biology and Southern Marine Science and Engineering Guangdong Laboratory (Zhuhai), School of Life Sciences, Sun Yat‑Sen University, Guangzhou 510275, PR China**

**^b^ State Key Laboratory of Desert and Oasis Ecology, Key Laboratory of Ecological Safety and Sustainable Development in Arid Lands, Xinjiang Institute of Ecology and Geography, Chinese Academy of Sciences, Urumqi 830011, PR China**

*** Corresponding author:**

**Lei Dong (donglei6@mail.sysu.edu.cn)**

**Tel: +86-13825005651; Fax: +86-20-84111727**

**Wen-Jun Li (liwenjun3@mail.sysu.edu.cn)**


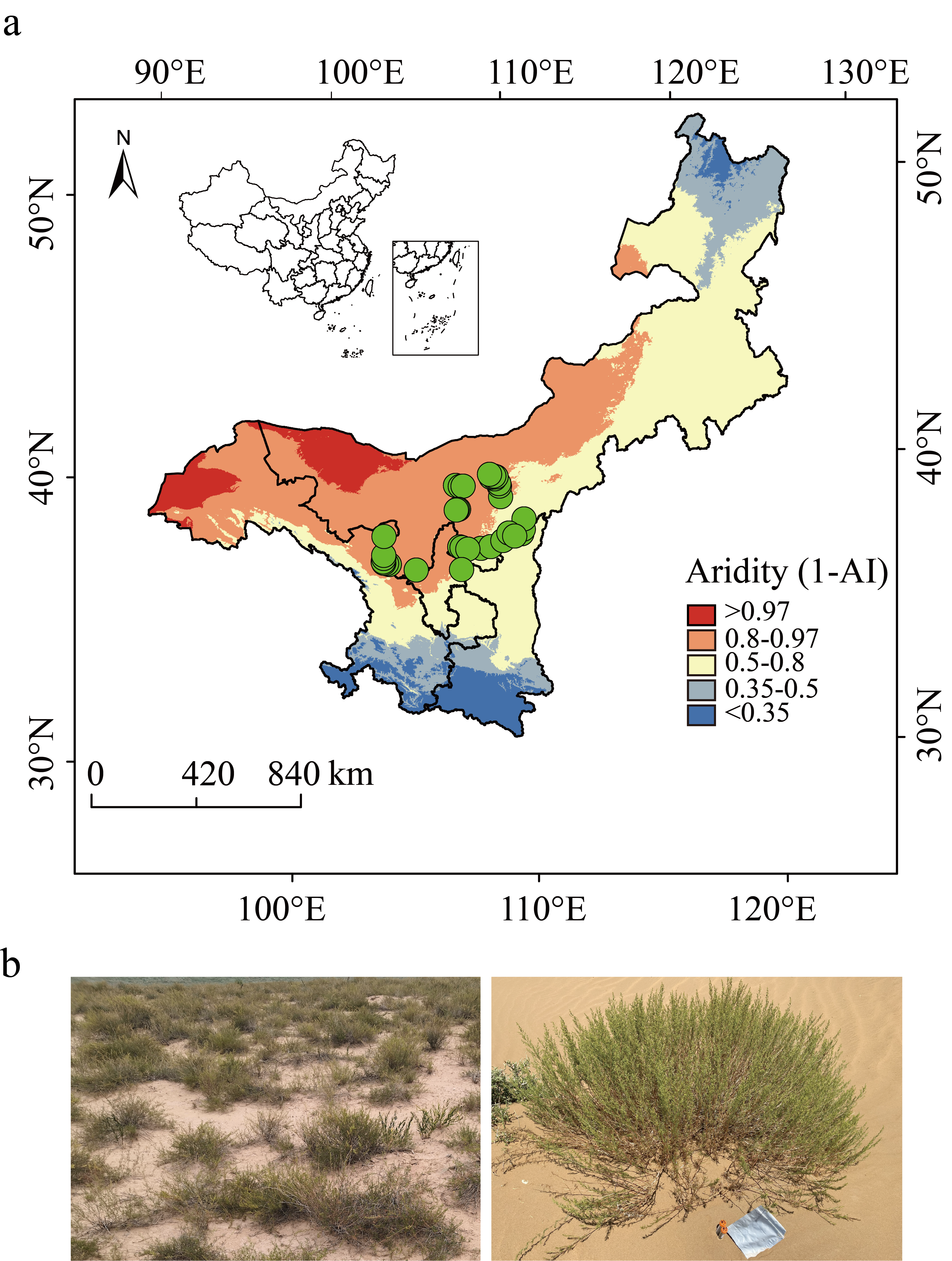


**Fig. S1. Distribution of sampling locations and collected samples in this study.** Green points represent sampling sites from this study, with each point indicating an independent sampling event.


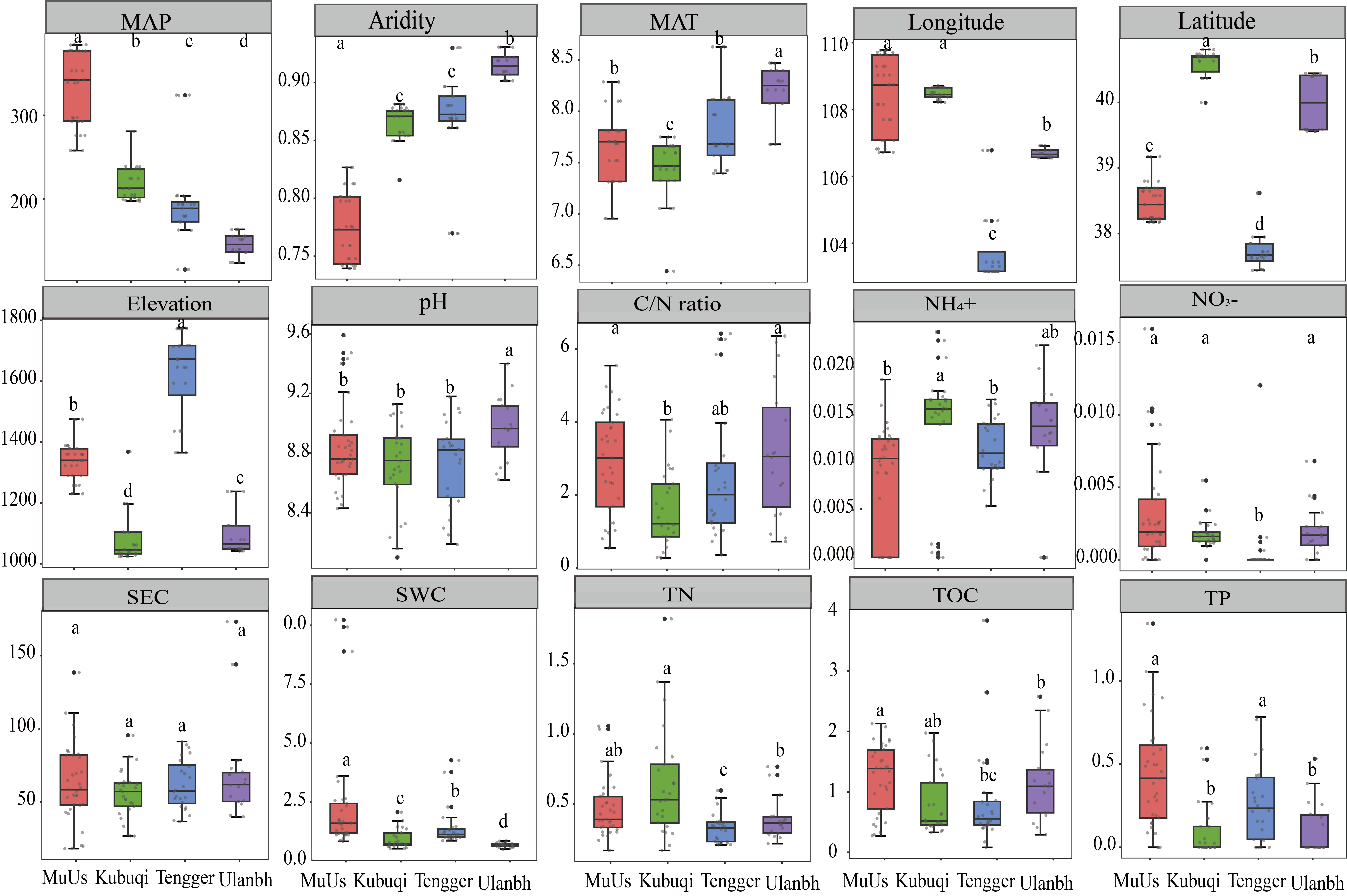


**Fig. S2. Differences in geographic, climatic, and physicochemical properties across different deserts.** Lowercase letters indicate significant differences among deserts (*P*_adj_ < 0.05, Benjamini-Hochberg corrected). Abbreviations: MAP, mean annual precipitation; MAT, mean annual temperature; NH_4_^+^, ammonium-nitrogen; NO_3_^-^, nitrate-nitrogen; SEC, soil electrical conductivity; SWC, soil water content; TN, total nitrogen; TP, total phosphorus; TOC, total organic carbon.





**Fig. S3. Differences in the relative abundance of the top ten phyla across different deserts.** **a**, **b** Differential abundance analysis at the phylum level comparing the abundant **a** and rare **b** bacterial subcommunities across soil samples from the four deserts. Lowercase letters indicate significant differences among the deserts (*P*_adj_ < 0.05, Benjamini-Hochberg corrected).


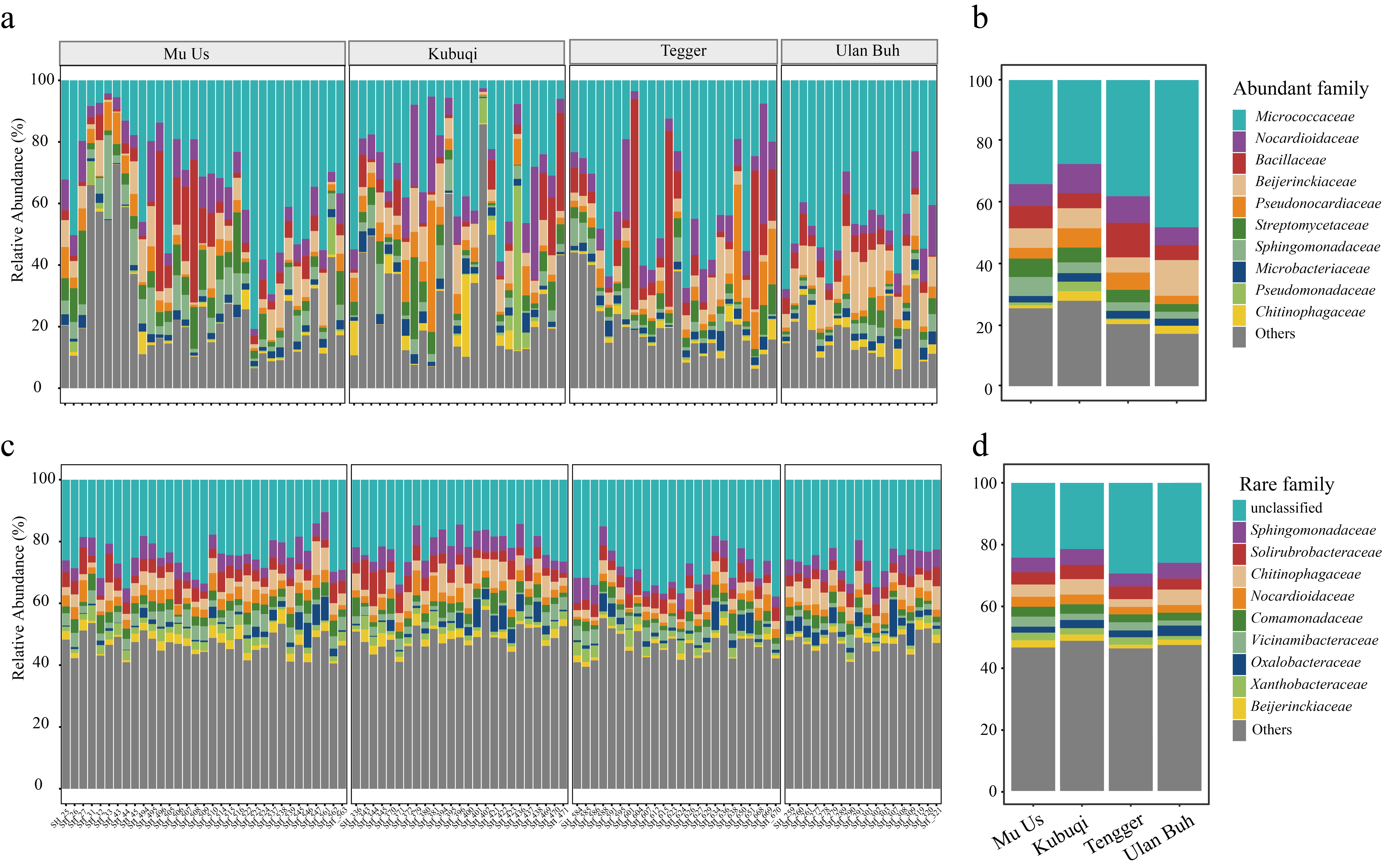


**Fig. S4. The composition of abundant and rare bacterial communities at the family level in the rhizosphere of *A. desertorum.*** **a**, **c** The composition of the top 10 families of abundant **a** and rare **c** bacterial subcommunities at all sampling points. **b**, **d** The average composition of the top 10 families of abundant **b** and rare bacterial subcommunities **d** across the four desert ecosystems.


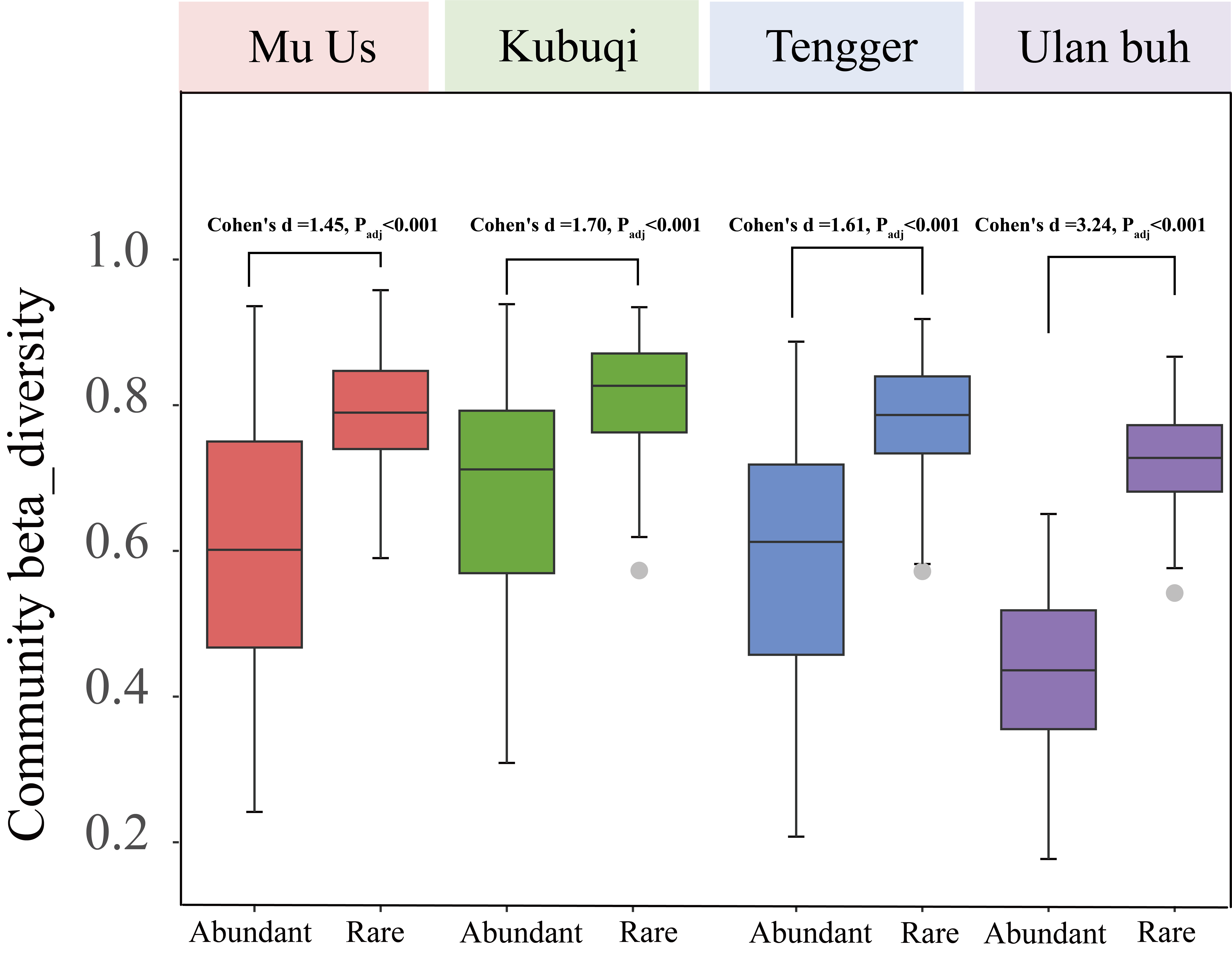


**Fig. S5. Comparison of *β*-diversity differences between abundant and rare subcommunities in the rhizosphere of *A. desertorum* across different deserts.** Difference of bacterial beta-diversity was estimated based on a Bray-Curtis distance matrix. Cohen’s d indicates the effect size.





**Figure S6. Composition and diversity of abundant and rare bacterial taxa in the rhizosphere of *Artemisia desertorum*, based on abundance thresholds (>0.1% for abundant and <0.01% for rare ZOTUs)**. **a**–**b** Taxonomic composition of abundant **a** and rare **b** subcommunities across different desert ecosystems. **c**–**d** *α*-diversity of abundant **c** and rare **d** subcommunities, measured by richness and Shannon index. Lowercase letters indicate significant differences among the deserts (*P*_adj_ < 0.05, Benjamini-Hochberg corrected). **e**–**f** NMDS ordination of abundant **e** and rare **f** subcommunities based on Bray–Curtis dissimilarity. Shaded ellipses represent 95% confidence intervals for each desert group. Group differences were evaluated using ANOSIM. **g** *β*-diversity of abundant and rare subcommunities across desert systems, calculated using Bray–Curtis distances. Cohen’s d indicates the effect size.


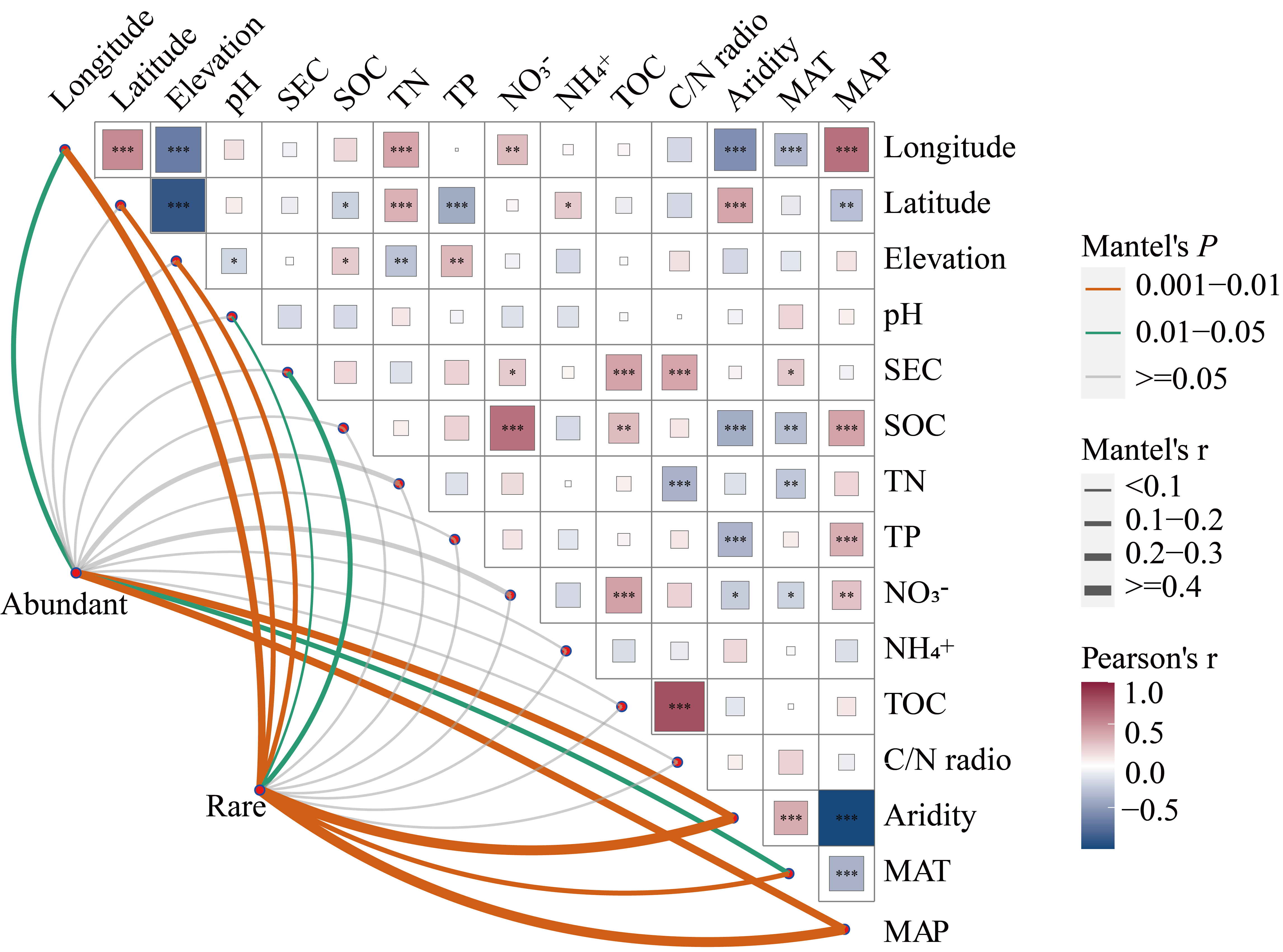


**Fig. S7. Pearson’s correlation coefficients between rare and abundant bacterial taxa and environmental factors based on Mantel tests.** Edge width corresponds to Mantel’s *r* value, and the color of the edge indicates the statistical significance based on 999 permutations. Pairwise correlations of these variables are displayed with circle size, while the color gradient indicates Pearso’s correlation coefficients. Squares with asterisks represent the significant levels at *P_adj_* < 0.05 (*), *P_adj_* < 0.01 (**), and *P_adj_* < 0.001 (***). Full abbreviations: MAP, mean annual precipitation; MAT, mean annual temperature; NH_4_^+^, ammonium-nitrogen; NO_3_^-^, nitrate-nitrogen; SEC, soil electrical conductivity; SWC, soil water content; TN, total nitrogen; TP, total phosphorus; TOC, total organic carbon.





**Fig. S8. Spearman correlation coefficients between the top 10 species at the phylum or family level and environmental factors.** **a**, **b** Correlations between abundant **a** and **b** rare bacterial subcommunities and environmental factors at the phylum level. **c**, **d** Correlations between abundant **c** and **d** rare bacterial subcommunities and environmental factors at the family level. Squares with asterisks represent the significant levels at *P_adj_* < 0.05 (*), *P_adj_* < 0.01 (**), and *P_adj_* < 0.001 (***). Full abbreviations: MAP, mean annual precipitation; MAT, mean annual temperature; NH_4_^+^, ammonium-nitrogen; NO_3_^-^, nitrate-nitrogen; SEC, soil electrical conductivity; SWC, soil water content; TN, total nitrogen; TP, total phosphorus; TOC, total organic carbon.


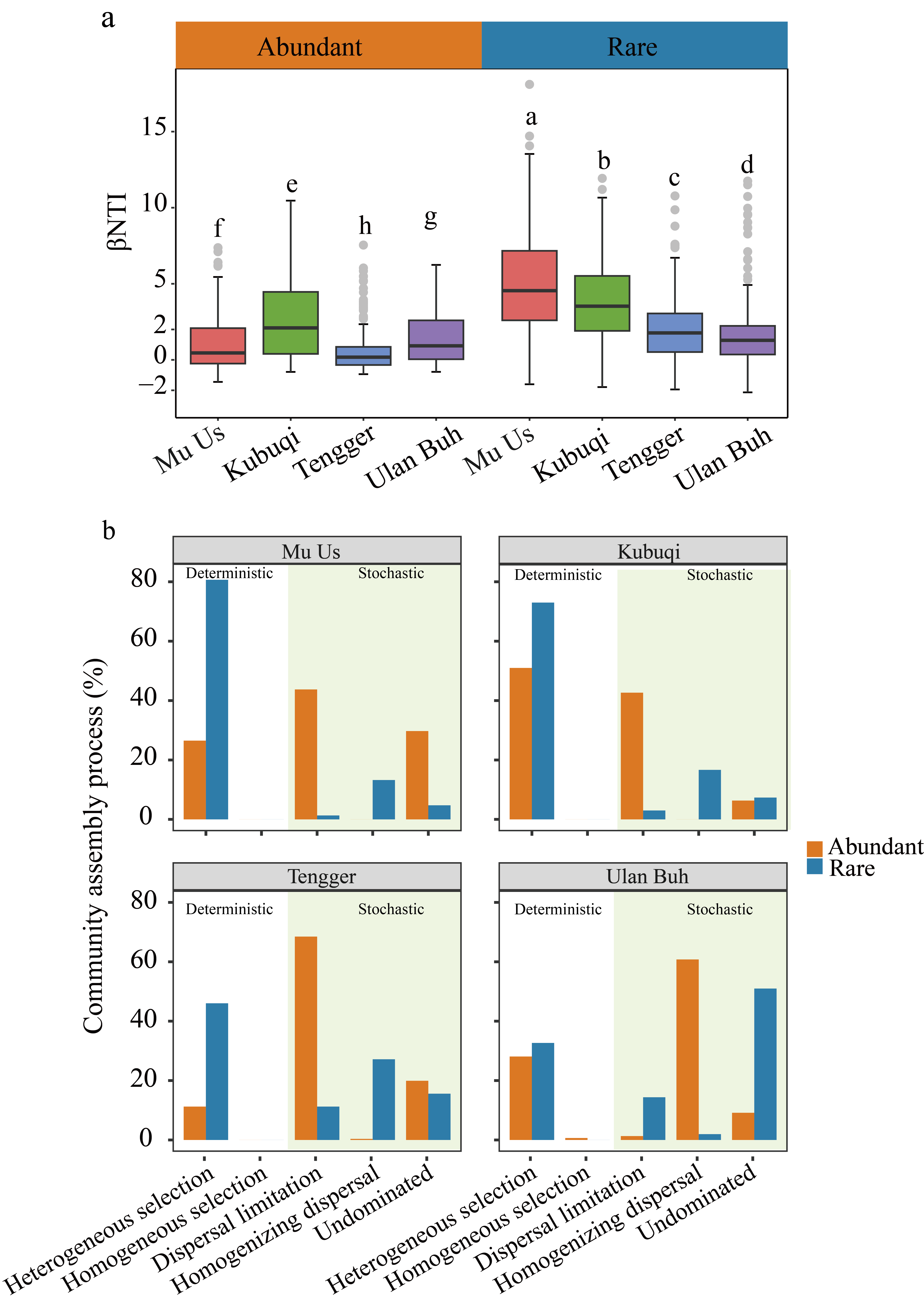


**Fig. S9. Ecological processes shaping the community assembly of rare and abundant taxa in *A. desertorum* rhizosphere ecosystems across different deserts.** **a** Significant differences were observed in the distribution of *β*NTI values between rare and abundant subcommunities across different deserts. Lowercase letters indicate significant differences among deserts (*P*_adj_ < 0.05, Benjamini-Hochberg corrected). **b** Relative contributions of different ecological processes to the assembly of rare and abundant subcommunities in *A. desertorum* rhizosphere soils in different deserts.





**Fig. S10. Functional prediction using PICRUSt2.** Histogram of predicted functional annotations at KEGG Level 1, showing relative abundances.

| **Table S1 Sampling Site Information.** Full abbreviations: MAP, mean annual precipitation; MAT, mean annual temperature; NH_4_^+^, ammonium-nitrogen; NO_3_^-^, nitrate-nitrogen; SEC, soil electrical conductivity; SWC, soil water content; TN, total nitrogen; TP, total phosphorus; TOC, total organic carbon. | | | | | | | | | | | | | | | | |
| --- | --- | --- | --- | --- | --- | --- | --- | --- | --- | --- | --- | --- | --- | --- | --- | --- |
| Sample ID | group | Longitude | Latitude | Elevation  (m) | pH | SEC  (μs/cm) | SWC  (%) | TN  (mg/g) | TP  (mg/g) | NO_3_^-^(mg/g) | NH_4_^+^  (mg/g) | TOC  (mg/g) | C/N ratio | Aridity index | MAT  (℃) | MAP  (mm) |
| SH_25 | Mu Us | 109.63 | 38.6 | 1230 | 9.47 | 138.5 | 0.860 | 0.273 | 0.587 | 0.001 | 0.013 | 0.459 | 1.678 | 0.748 | 7.704 | 377 |
| SH_26 | Mu Us | 109.63 | 38.6 | 1230 | 9.59 | 42.1 | 0.938 | 0.242 | 0.455 | 0.000 | 0.012 | 0.298 | 1.232 | 0.748 | 7.704 | 377 |
| SH_27 | Mu Us | 109.63 | 38.6 | 1230 | 9.43 | 43.1 | 1.538 | 1.034 | 0.487 | 0.000 | 0.012 | 0.561 | 0.543 | 0.748 | 7.704 | 377 |
| SH_31 | Mu Us | 109.71 | 38.8 | 1290 | 9.4 | 18.32 | 2.594 | 0.707 | 0.196 | 0.000 | 0.013 | 0.719 | 1.018 | 0.739 | 7.313 | 384 |
| SH_32 | Mu Us | 109.71 | 38.8 | 1290 | 9.21 | 20.05 | 2.426 | 0.508 | 0.084 | 0.000 | 0.010 | 0.488 | 0.960 | 0.739 | 7.313 | 384 |
| SH_33 | Mu Us | 109.71 | 38.8 | 1290 | 9.01 | 18.38 | 2.637 | 0.391 | 0.118 | 0.010 | 0.010 | 1.695 | 4.333 | 0.739 | 7.313 | 384 |
| SH_43 | Mu Us | 109.77 | 39.2 | 1340 | 9.02 | 20.42 | 3.373 | 1.055 | 0.000 | 0.006 | 0.012 | 1.746 | 1.655 | 0.742 | 6.954 | 377 |
| SH_44 | Mu Us | 109.77 | 39.2 | 1340 | 8.79 | 29.3 | 1.599 | 0.621 | 0.086 | 0.004 | 0.014 | 0.636 | 1.025 | 0.742 | 6.954 | 377 |
| SH_45 | Mu Us | 109.77 | 39.2 | 1340 | 8.92 | 45.1 | 3.588 | 0.346 | 0.143 | 0.002 | 0.013 | 0.425 | 1.228 | 0.742 | 6.954 | 377 |
| SH_259 | Ulan Buh | 106.55 | 40.4 | 1042 | 9.4 | 78.7 | 0.825 | 0.405 | 0.180 | 0.002 | 0.000 | 2.573 | 6.358 | 0.931 | 8.471 | 124 |
| SH_260 | Ulan Buh | 106.55 | 40.4 | 1042 | 9.25 | 50.3 | 0.542 | 0.707 | 0.383 | 0.001 | 0.014 | 0.656 | 0.928 | 0.931 | 8.471 | 124 |
| SH_261 | Ulan Buh | 106.55 | 40.4 | 1042 | 9.16 | 61.6 | 0.695 | 0.261 | 0.532 | 0.004 | 0.012 | 0.817 | 3.128 | 0.931 | 8.471 | 124 |
| SH_277 | Ulan Buh | 106.79 | 40.4 | 1050 | 9.12 | 50.9 | 0.708 | 0.378 | 0.138 | 0.000 | 0.013 | 0.984 | 2.606 | 0.922 | 8.396 | 137 |
| SH_278 | Ulan Buh | 106.79 | 40.4 | 1050 | 9.1 | 60.5 | 0.646 | 0.411 | 0.199 | 0.001 | 0.020 | 1.298 | 3.155 | 0.922 | 8.396 | 137 |
| SH_279 | Ulan Buh | 106.79 | 40.4 | 1050 | 8.95 | 73.1 | 0.525 | 0.218 | 0.258 | 0.002 | 0.013 | 1.145 | 5.246 | 0.922 | 8.396 | 137 |
| SH_289 | Ulan Buh | 106.92 | 40.4 | 1049 | 9.16 | 40.9 | 0.620 | 0.565 | 0.000 | 0.001 | 0.016 | 0.408 | 0.722 | 0.919 | 8.296 | 140 |
| SH_290 | Ulan Buh | 106.92 | 40.4 | 1049 | 8.97 | 70.5 | 0.761 | 0.284 | 0.000 | 0.000 | 0.012 | 1.656 | 5.825 | 0.919 | 8.296 | 140 |
| SH_291 | Ulan Buh | 106.92 | 40.4 | 1049 | 8.87 | 65.5 | 0.686 | 0.414 | 0.270 | 0.000 | 0.014 | 0.612 | 1.479 | 0.919 | 8.296 | 140 |
| SH_301 | Ulan Buh | 106.68 | 39.6 | 1079 | 8.62 | 173.1 | 0.738 | 0.379 | 0.000 | 0.003 | 0.017 | 2.346 | 6.188 | 0.909 | 8.208 | 152 |
| SH_302 | Ulan Buh | 106.68 | 39.6 | 1079 | 8.66 | 144 | 0.650 | 0.352 | 0.000 | 0.001 | 0.022 | 1.083 | 3.078 | 0.909 | 8.208 | 152 |
| SH_303 | Ulan Buh | 106.68 | 39.6 | 1079 | 8.97 | 69.1 | 0.564 | 0.766 | 0.000 | 0.002 | 0.000 | 1.780 | 2.323 | 0.909 | 8.208 | 152 |
| SH_307 | Ulan Buh | 106.63 | 39.6 | 1125 | 8.84 | 53.8 | 0.484 | 0.354 | 0.000 | 0.004 | 0.009 | 0.291 | 0.822 | 0.907 | 8.079 | 156 |
| SH_308 | Ulan Buh | 106.63 | 39.6 | 1125 | 8.96 | 50.3 | 0.623 | 0.330 | 0.000 | 0.002 | 0.000 | 0.473 | 1.436 | 0.907 | 8.079 | 156 |
| SH_309 | Ulan Buh | 106.63 | 39.6 | 1125 | 8.99 | 40 | 0.596 | 0.289 | 0.000 | 0.002 | 0.013 | 0.654 | 2.262 | 0.907 | 8.079 | 156 |
| SH_319 | Ulan Buh | 106.57 | 39.6 | 1238 | 8.7 | 68.8 | 0.832 | 0.390 | 0.000 | 0.007 | 0.017 | 1.181 | 3.027 | 0.901 | 7.679 | 164 |
| SH_320 | Ulan Buh | 106.57 | 39.6 | 1238 | 8.73 | 62.5 | 0.666 | 0.307 | 0.000 | 0.002 | 0.016 | 1.388 | 4.515 | 0.901 | 7.679 | 164 |
| SH_321 | Ulan Buh | 106.57 | 39.6 | 1238 | 8.85 | 48.8 | 0.707 | 0.271 | 0.000 | 0.000 | 0.016 | 1.099 | 4.053 | 0.901 | 7.679 | 164 |
| SH_336 | Kubuqi | 108.71 | 40 | 1368 | 9.13 | 53.8 | 0.685 | 0.836 | 0.000 | 0.001 | 0.000 | 0.349 | 0.418 | 0.816 | 6.442 | 281 |
| SH_343 | Kubuqi | 108.65 | 40.4 | 1197 | 9.07 | 62.9 | 1.441 | 0.742 | 0.000 | 0.002 | 0.018 | 1.529 | 2.062 | 0.850 | 7.054 | 239 |
| SH_344 | Kubuqi | 108.65 | 40.4 | 1197 | 9.05 | 63.2 | 1.689 | 0.588 | 0.000 | 0.002 | 0.001 | 1.598 | 2.716 | 0.850 | 7.054 | 239 |
| SH_345 | Kubuqi | 108.65 | 40.4 | 1197 | 8.78 | 81.1 | 2.057 | 0.901 | 0.000 | 0.003 | 0.001 | 1.972 | 2.190 | 0.850 | 7.054 | 239 |
| SH_370 | Kubuqi | 108.65 | 40.5 | 1104 | 9.06 | 26.9 | 0.612 | 1.241 | 0.000 | 0.002 | 0.000 | 0.330 | 0.266 | 0.854 | 7.467 | 236 |
| SH_371 | Kubuqi | 108.65 | 40.5 | 1104 | 8.9 | 33.6 | 0.671 | 1.819 | 0.274 | 0.001 | 0.015 | 0.534 | 0.294 | 0.854 | 7.467 | 236 |
| SH_372 | Kubuqi | 108.65 | 40.5 | 1104 | 8.98 | 27 | 0.506 | 0.306 | 0.000 | 0.001 | 0.016 | 0.425 | 1.388 | 0.854 | 7.467 | 236 |
| SH_379 | Kubuqi | 108.45 | 40.6 | 1062 | 8.87 | 57.3 | 0.720 | 1.057 | 0.000 | 0.003 | 0.017 | 1.150 | 1.088 | 0.871 | 7.662 | 213 |
| SH_380 | Kubuqi | 108.45 | 40.6 | 1062 | 8.1 | 64.2 | 0.772 | 0.529 | 0.000 | 0.002 | 0.021 | 0.641 | 1.212 | 0.871 | 7.662 | 213 |
| SH_381 | Kubuqi | 108.45 | 40.6 | 1062 | 8.16 | 59.8 | 0.997 | 0.784 | 0.262 | 0.002 | 0.023 | 1.279 | 1.632 | 0.871 | 7.662 | 213 |
| SH_394 | Kubuqi | 108.28 | 40.7 | 1046 | 8.23 | 49.2 | 0.691 | 0.647 | 0.000 | 0.002 | 0.017 | 0.624 | 0.965 | 0.881 | 7.750 | 198 |
| SH_395 | Kubuqi | 108.28 | 40.7 | 1046 | 8.31 | 62.3 | 0.775 | 0.340 | 0.175 | 0.000 | 0.001 | 0.782 | 2.301 | 0.881 | 7.750 | 198 |
| SH_396 | Kubuqi | 108.28 | 40.7 | 1046 | 8.33 | 42.1 | 0.716 | 0.411 | 0.026 | 0.002 | 0.014 | 0.473 | 1.152 | 0.881 | 7.750 | 198 |
| SH_400 | Kubuqi | 108.37 | 40.7 | 1038 | 8.57 | 61.2 | 0.829 | 0.650 | 0.173 | 0.001 | 0.016 | 0.366 | 0.564 | 0.878 | 7.662 | 202 |
| SH_401 | Kubuqi | 108.37 | 40.7 | 1038 | 8.65 | 58.2 | 0.514 | 1.370 | 0.000 | 0.001 | 0.015 | 0.400 | 0.292 | 0.878 | 7.662 | 202 |
| SH_402 | Kubuqi | 108.37 | 40.7 | 1038 | 8.59 | 72.5 | 1.080 | 0.576 | 0.030 | 0.005 | 0.000 | 0.435 | 0.755 | 0.878 | 7.662 | 202 |
| SH_421 | Kubuqi | 108.51 | 40.7 | 1024 | 8.63 | 51.6 | 1.170 | 0.532 | 0.000 | 0.002 | 0.015 | 0.454 | 0.854 | 0.857 | 7.325 | 225 |
| SH_422 | Kubuqi | 108.51 | 40.7 | 1024 | 8.71 | 49.5 | 0.546 | 0.522 | 0.000 | 0.001 | 0.024 | 0.449 | 0.861 | 0.857 | 7.325 | 225 |
| SH_423 | Kubuqi | 108.51 | 40.7 | 1024 | 8.68 | 45.2 | 0.589 | 0.430 | 0.123 | 0.002 | 0.017 | 0.506 | 1.179 | 0.857 | 7.325 | 225 |
| SH_436 | Kubuqi | 108.42 | 40.7 | 1033 | 8.69 | 52 | 0.681 | 0.296 | 0.025 | 0.001 | 0.015 | 0.521 | 1.760 | 0.876 | 7.596 | 205 |
| SH_437 | Kubuqi | 108.42 | 40.7 | 1033 | 8.8 | 47.2 | 0.672 | 0.205 | 0.000 | 0.002 | 0.016 | 0.514 | 2.505 | 0.876 | 7.596 | 205 |
| SH_438 | Kubuqi | 108.42 | 40.7 | 1033 | 8.75 | 38.8 | 0.616 | 0.170 | 0.051 | 0.001 | 0.017 | 0.481 | 2.820 | 0.876 | 7.596 | 205 |
| SH_469 | Kubuqi | 108.22 | 40.8 | 1029 | 8.91 | 71.1 | 1.177 | 0.291 | 0.526 | 0.002 | 0.016 | 0.795 | 2.729 | 0.875 | 7.433 | 200 |
| SH_470 | Kubuqi | 108.22 | 40.8 | 1029 | 8.86 | 95.7 | 1.421 | 0.367 | 0.595 | 0.002 | 0.014 | 1.375 | 3.750 | 0.875 | 7.433 | 200 |
| SH_471 | Kubuqi | 108.22 | 40.8 | 1029 | 8.9 | 79.1 | 1.346 | 0.453 | 0.125 | 0.001 | 0.017 | 1.842 | 4.063 | 0.875 | 7.433 | 200 |
| SH_494 | Mu Us | 107.69 | 38.2 | 1360 | 8.85 | 58.5 | 1.095 | 0.333 | 0.303 | 0.002 | 0.019 | 1.415 | 4.248 | 0.801 | 7.808 | 297 |
| SH_495 | Mu Us | 107.69 | 38.2 | 1360 | 8.74 | 54.5 | 1.172 | 0.302 | 0.000 | 0.003 | 0.012 | 1.393 | 4.615 | 0.801 | 7.808 | 297 |
| SH_496 | Mu Us | 107.69 | 38.2 | 1360 | 8.76 | 82.1 | 1.733 | 0.381 | 0.414 | 0.002 | 0.016 | 1.149 | 3.013 | 0.801 | 7.808 | 297 |
| SH_505 | Mu Us | 108.15 | 38.2 | 1378 | 8.65 | 94.6 | 1.098 | 0.553 | 0.192 | 0.002 | 0.011 | 2.130 | 3.853 | 0.759 | 7.817 | 353 |
| SH_506 | Mu Us | 108.15 | 38.2 | 1378 | 8.71 | 64.8 | 1.167 | 0.493 | 0.322 | 0.003 | 0.000 | 2.076 | 4.208 | 0.759 | 7.817 | 353 |
| SH_507 | Mu Us | 108.15 | 38.2 | 1378 | 8.66 | 80.4 | 1.174 | 0.572 | 1.344 | 0.005 | 0.011 | 1.340 | 2.341 | 0.759 | 7.817 | 353 |
| SH_508 | Mu Us | 108.74 | 38.4 | 1340 | 8.76 | 48 | 1.586 | 0.510 | 0.897 | 0.001 | 0.014 | 1.797 | 3.526 | 0.775 | 7.704 | 339 |
| SH_509 | Mu Us | 108.74 | 38.4 | 1340 | 8.53 | 48.3 | 1.723 | 0.457 | 0.280 | 0.003 | 0.000 | 1.082 | 2.366 | 0.775 | 7.704 | 339 |
| SH_510 | Mu Us | 108.74 | 38.4 | 1340 | 8.73 | 53.4 | 2.125 | 0.279 | 0.496 | 0.002 | 0.014 | 1.386 | 4.964 | 0.775 | 7.704 | 339 |
| SH_514 | Mu Us | 109.03 | 38.7 | 1388 | 8.45 | 102.8 | 9.933 | 0.555 | 0.613 | 0.016 | 0.009 | 1.525 | 2.746 | 0.773 | 7.317 | 342 |
| SH_515 | Mu Us | 109.03 | 38.7 | 1388 | 8.43 | 110.8 | 8.889 | 0.616 | 0.917 | 0.010 | 0.012 | 1.581 | 2.568 | 0.773 | 7.317 | 342 |
| SH_516 | Mu Us | 109.03 | 38.7 | 1388 | 8.49 | 68.6 | 10.232 | 0.443 | 0.177 | 0.008 | 0.000 | 1.768 | 3.992 | 0.773 | 7.317 | 342 |
| SH_522 | Mu Us | 109.3 | 38.6 | 1258 | 8.51 | 82.1 | 2.325 | 0.430 | 0.275 | 0.001 | 0.006 | 1.649 | 3.836 | 0.743 | 7.688 | 377 |
| SH_523 | Mu Us | 109.3 | 38.6 | 1258 | 8.65 | 62.4 | 1.649 | 0.486 | 0.000 | 0.002 | 0.009 | 1.510 | 3.110 | 0.743 | 7.688 | 377 |
| SH_524 | Mu Us | 109.3 | 38.6 | 1258 | 8.75 | 84.4 | 2.552 | 0.804 | 0.560 | 0.001 | 0.010 | 1.863 | 2.317 | 0.743 | 7.688 | 377 |
| SH_537 | Mu Us | 106.72 | 38.2 | 1322 | 8.74 | 69.8 | 1.191 | 0.386 | 0.147 | 0.002 | 0.000 | 1.396 | 3.617 | 0.827 | 8.288 | 258 |
| SH_538 | Mu Us | 106.72 | 38.2 | 1322 | 8.63 | 85.2 | 1.176 | 0.300 | 0.514 | 0.002 | 0.010 | 1.045 | 3.487 | 0.827 | 8.288 | 258 |
| SH_539 | Mu Us | 106.72 | 38.2 | 1322 | 8.84 | 54.4 | 1.096 | 0.320 | 0.820 | 0.000 | 0.009 | 1.109 | 3.462 | 0.827 | 8.288 | 258 |
| SH_545 | Mu Us | 106.82 | 38.2 | 1360 | 8.88 | 54.8 | 1.347 | 0.381 | 0.639 | 0.000 | 0.010 | 1.841 | 4.827 | 0.813 | 8.100 | 276 |
| SH_546 | Mu Us | 106.82 | 38.2 | 1360 | 8.95 | 48.6 | 1.094 | 0.338 | 0.656 | 0.002 | 0.000 | 1.875 | 5.546 | 0.813 | 8.100 | 276 |
| SH_547 | Mu Us | 106.82 | 38.2 | 1360 | 8.76 | 82.8 | 1.160 | 0.372 | 0.384 | 0.009 | 0.000 | 0.967 | 2.602 | 0.813 | 8.100 | 276 |
| SH_561 | Mu Us | 107.08 | 38.2 | 1475 | 8.84 | 57.4 | 1.293 | 0.301 | 1.054 | 0.005 | 0.000 | 1.323 | 4.391 | 0.798 | 7.521 | 293 |
| SH_562 | Mu Us | 107.08 | 38.2 | 1475 | 8.82 | 70.7 | 0.818 | 0.171 | 0.858 | 0.001 | 0.000 | 0.325 | 1.906 | 0.798 | 7.521 | 293 |
| SH_563 | Mu Us | 107.08 | 38.2 | 1475 | 8.87 | 59.9 | 1.662 | 0.340 | 0.495 | 0.001 | 0.000 | 0.272 | 0.798 | 0.798 | 7.521 | 293 |
| SH_584 | Tengger | 106.78 | 37.5 | 1436 | 8.79 | 91.4 | 1.323 | 0.501 | 0.434 | 0.001 | 0.016 | 0.454 | 0.905 | 0.770 | 8.629 | 324 |
| SH_585 | Tengger | 106.78 | 37.5 | 1436 | 9.01 | 58.1 | 0.990 | 0.352 | 0.219 | 0.001 | 0.013 | 0.430 | 1.222 | 0.770 | 8.629 | 324 |
| SH_586 | Tengger | 106.78 | 37.5 | 1436 | 9.18 | 83.6 | 1.095 | 0.340 | 0.416 | 0.000 | 0.014 | 0.636 | 1.872 | 0.770 | 8.629 | 324 |
| SH_588 | Tengger | 104.67 | 37.4 | 1717 | 8.3 | 87.2 | 1.039 | 0.210 | 0.306 | 0.000 | 0.014 | 0.834 | 3.970 | 0.861 | 7.662 | 204 |
| SH_591 | Tengger | 104.67 | 37.4 | 1717 | 8.25 | 45.1 | 0.875 | 0.229 | 0.250 | 0.000 | 0.014 | 0.082 | 0.357 | 0.861 | 7.662 | 204 |
| SH_595 | Tengger | 104.67 | 37.4 | 1717 | 8.45 | 47 | 1.009 | 0.234 | 0.765 | 0.000 | 0.015 | 0.172 | 0.733 | 0.861 | 7.662 | 204 |
| SH_601 | Tengger | 103.44 | 37.6 | 1772 | 8.19 | 78.3 | 1.302 | 0.252 | 0.782 | 0.000 | 0.017 | 0.673 | 2.666 | 0.869 | 7.425 | 194 |
| SH_604 | Tengger | 103.44 | 37.6 | 1772 | 8.35 | 69.5 | 2.279 | 0.321 | 0.156 | 0.002 | 0.010 | 0.984 | 3.070 | 0.869 | 7.425 | 194 |
| SH_607 | Tengger | 103.44 | 37.6 | 1772 | 8.48 | 60.6 | 1.180 | 0.260 | 0.296 | 0.000 | 0.016 | 1.520 | 5.851 | 0.869 | 7.425 | 194 |
| SH_612 | Tengger | 103.31 | 37.7 | 1774 | 8.51 | 53 | 1.005 | 0.222 | 0.154 | 0.000 | 0.012 | 0.607 | 2.734 | 0.869 | 7.396 | 194 |
| SH_615 | Tengger | 103.31 | 37.7 | 1774 | 8.56 | 54 | 1.125 | 0.312 | 0.432 | 0.000 | 0.015 | 0.400 | 1.283 | 0.869 | 7.396 | 194 |
| SH_623 | Tengger | 103.16 | 37.7 | 1713 | 9.1 | 82.2 | 1.833 | 0.421 | 0.000 | 0.000 | 0.014 | 2.644 | 6.274 | 0.872 | 7.571 | 189 |
| SH_624 | Tengger | 103.16 | 37.7 | 1713 | 8.85 | 89.1 | 3.759 | 0.597 | 0.216 | 0.012 | 0.007 | 3.834 | 6.422 | 0.872 | 7.571 | 189 |
| SH_626 | Tengger | 103.15 | 37.7 | 1673 | 8.9 | 52.4 | 1.193 | 0.221 | 0.104 | 0.000 | 0.011 | 0.476 | 2.149 | 0.880 | 7.683 | 180 |
| SH_627 | Tengger | 103.15 | 37.7 | 1673 | 8.82 | 57.4 | 0.999 | 0.211 | 0.587 | 0.000 | 0.005 | 0.590 | 2.802 | 0.880 | 7.683 | 180 |
| SH_629 | Tengger | 103.15 | 37.7 | 1673 | 8.89 | 49.8 | 1.239 | 0.379 | 0.273 | 0.000 | 0.009 | 0.466 | 1.231 | 0.880 | 7.683 | 180 |
| SH_634 | Tengger | 103.16 | 37.8 | 1646 | 8.82 | 36.8 | 0.845 | 0.348 | 0.558 | 0.000 | 0.008 | 0.517 | 1.486 | 0.888 | 7.967 | 173 |
| SH_636 | Tengger | 103.16 | 37.8 | 1646 | 8.73 | 52.3 | 1.065 | 0.222 | 0.000 | 0.000 | 0.010 | 0.520 | 2.338 | 0.888 | 7.967 | 173 |
| SH_638 | Tengger | 103.16 | 37.8 | 1646 | 8.76 | 46.1 | 4.265 | 0.337 | 0.000 | 0.000 | 0.010 | 0.488 | 1.446 | 0.888 | 7.967 | 173 |
| SH_650 | Tengger | 103.16 | 37.9 | 1593 | 8.84 | 37.6 | 1.448 | 0.370 | 0.047 | 0.000 | 0.009 | 0.405 | 1.095 | 0.897 | 8.133 | 163 |
| SH_651 | Tengger | 103.16 | 37.9 | 1593 | 8.85 | 40.7 | 1.410 | 0.372 | 0.327 | 0.000 | 0.009 | 0.383 | 1.031 | 0.897 | 8.133 | 163 |
| SH_668 | Tengger | 103.16 | 38.6 | 1365 | 8.87 | 74.4 | 0.893 | 0.543 | 0.048 | 0.000 | 0.011 | 0.858 | 1.579 | 0.930 | 8.113 | 116 |
| SH_669 | Tengger | 103.16 | 38.6 | 1365 | 9.06 | 66.8 | 0.929 | 0.359 | 0.000 | 0.000 | 0.008 | 0.792 | 2.204 | 0.930 | 8.113 | 116 |
| SH_670 | Tengger | 103.16 | 38.6 | 1365 | 9.07 | 71.3 | 0.928 | 0.236 | 0.042 | 0.000 | 0.009 | 1.478 | 6.269 | 0.930 | 8.113 | 116 |

**Table S2 General description of ZOTUs datasets.**

|  | **ASV numbers** | **sequence** | **Chao1** | **ACE** |
| --- | --- | --- | --- | --- |
| All | 9796 | 1068658 | 3989±70 | 4147±75 |
| Abundant | 217(2.22%) | 263379(24.65%) |  |  |
| Rare | 5706(58.25%) | 216532(20.26%) |  |  |

**Table S3 Observed phyla and their relative abundance from abundant bacterial subcommunities and rare bacterial subcommunities.**

| **Phylum** | **Abundant relative abundance** |  | **Rare relative abundance** |
| --- | --- | --- | --- |
| *Actinomycetota* | 58.58 |  | 32.7 |
| *Pseudomonadota* | 22.14 |  | 30.99 |
| *Bacillota* | 10.19 |  | 1.73 |
| *Bacteroidota* | 4.23 |  | 6.61 |
| *Acidobacteriota* | 2.29 |  | 10.1 |
| *Patescibacteria* | 1.25 |  | 0.71 |
| *Gemmatimonadota* | 0.86 |  | 2.28 |
| *Chloroflexota* | 0.15 |  | 8.61 |
| *Myxococcota* | 0.13 |  | 2.32 |
| *Verrucomicrobiota* | 0.1 |  | 1.58 |
| *Deinococcota* | 0.08 |  | 0.12 |
| Unclassified_bacteria |  |  | 0.61 |
| *Nitrospirota* |  |  | 0.4 |
| *Cyanobacteriota* |  |  | 0.3 |
| *Methylomirabilota* |  |  | 0.2 |
| *Planctomycetota* |  |  | 0.18 |
| *Bdellovibrionota* |  |  | 0.13 |
| RCP2-54 |  |  | 0.09 |
| *Entotheonellaeota* |  |  | 0.08 |
| WPS-2 |  |  | 0.047 |
| *Abditibacteriota* |  |  | 0.045 |
| *Sumerlaeota* |  |  | 0.036 |
| *Desulfobacterota* |  |  | 0.032 |
| *Armatimonadota* |  |  | 0.024 |
| *Elusimicrobiota* |  |  | 0.021 |
| MBNT15 |  |  | 0.014 |
| *Dependentiae* |  |  | 0.014 |
| WS2 |  |  | 0.009 |
| *Latescibacterota* |  |  | 0.009 |
| *Fibrobacterota* |  |  | 0.009 |

| **Table S4 Taxonomic composition and relative abundances of phyla in abundant and rare subcommunities (cutoffs: >0.1% and <0.01%)** | | |
| --- | --- | --- |
| **Phylum** | **Abundant relative abundance** | **Rare relative abundance** |
| *Actinomycetota* | 59.839 | 30.667 |
| *Pseudomonadota* | 28.435 | 27.469 |
| *Acidobacteriota* | 1.286 | 10.181 |
| *Bacteroidota* | 1.265 | 9.051 |
| *Chloroflexota* | 1.602 | 7.924 |
| *Bacillota* | 5.493 | 4.468 |
| *Gemmatimonadota* | 1.578 | 2.743 |
| *Nitrospirota* | 0.502 | 0.356 |
| *Myxococcota* |  | 2.252 |
| *Patescibacteria* |  | 1.602 |
| *Verrucomicrobiota* |  | 1.545 |
| *Unclassified_bacteria* |  | 0.606 |
| *Methylomirabilota* |  | 0.154 |
| *Cyanobacteriota* |  | 0.159 |
| *Planctomycetota* |  | 0.139 |
| *Bdellovibrionota* |  | 0.123 |
| *Deinococcota* |  | 0.100 |
| *Entotheonellaeota* |  | 0.087 |
| *Desulfobacterota* |  | 0.069 |
| RCP2-54 |  | 0.065 |
| WPS-2 |  | 0.034 |
| *Abditibacteriota* |  | 0.031 |
| *Sumerlaeota* |  | 0.027 |
| *Fusobacteriota* |  | 0.020 |
| *Latescibacterota* |  | 0.016 |
| *Armatimonadota* |  | 0.017 |
| *Dependentiae* |  | 0.017 |
| WS2 |  | 0.013 |
| *Dependentiae* |  | 0.015 |
| *Deferribacterota* |  | 0.014 |
| GAL15 |  | 0.011 |
| MBNT15 |  | 0.011 |
| *Synergistota* |  | 0.011 |
| *Fibrobacterota* |  | 0.006 |

**Table S5 Mantel tests of environmental factors against the abundant and rare bacteria taxa in desert ecosystems.** Full abbreviations: MAP, mean annual precipitation; MAT, mean annual temperature; NH_4_^+^, ammonium-nitrogen; NO_3_^-^, nitrate-nitrogen; SEC, soil electrical conductivity; SWC, soil water content; TN, total nitrogen; TP, total phosphorus; TOC, total organic carbon.

| **Environmental factor** | **Abundant** | | | **Rare** | | |
| --- | --- | --- | --- | --- | --- | --- |
|  | R | *P*-value | *P_adj_*-value | R | *P*-value | *P_adj_*-value |
| Longitude | 0.105 | 0.031 | 0.085 | **0.256(**)** | **0.001** | **0.0043** |
| Latitude | 0.043 | 0.061 | 0.11 | **0.146(**)** | **0.001** | **0.0043** |
| Elevation | -0.022 | 0.679 | 0.7 | **0.122(*)** | **0.003** | **0.011** |
| pH | 0.055 | 0.186 | 0.25 | 0.08 | 0.042 | 0.105 |
| SEC | 0.065 | 0.164 | 0.25 | **0.114(*)** | **0.021** | **0.043** |
| SWC | 0.058 | 0.226 | 0.28 | 0.048 | 0.18 | 0.253 |
| TN | 0.124 | 0.056 | 0.11 | 0.087 | 0.054 | 0.112 |
| TP | -0.06 | 0.857 | 0.86 | 0.017 | 0.326 | 0.391 |
| NO_3_^-^ | 0.1 | 0.086 | 0.15 | 0.067 | 0.102 | 0.17 |
| NH_4_^+^ | -0.007 | 0.539 | 0.62 | -0.008 | 0.564 | 0.627 |
| TOC | 0.059 | 0.167 | 0.25 | 0.075 | 0.053 | 0.112 |
| C/N ratio | -0.018 | 0.62 | 0.66 | 0.032 | 0.194 | 0.253 |
| Aridity | **0.206(**)** | **0.001** | **0.004** | **0.46(**)** | **0.001** | **0.0043** |
| MAT | **0.120(*)** | **0.017** | **0.046** | **0.183(**)** | **0.001** | **0.0043** |
| MAP | **0.214(***)** | **0.001** | **0.001** | **0.472(**)** | **0.001** | **0.0043** |

**Table S6 The topological features of empirical network and random network.**

|  | **Empirical network** | | | | | | |  | **Random network** | | | |
| --- | --- | --- | --- | --- | --- | --- | --- | --- | --- | --- | --- | --- |
|  | Nodes | Edges | Modularity | Average clustering coefficient | Network diameter | Average path length | Average degree |  | Modularity (SD) | Average clustering coefficient (SD) | Average path length (SD) | Small-word coefficient (SD) |
| **Whole network** | 320 | 1557 | 0.42 | 0.39 | 13 | 3.52 | 9.73 |  | 0.25  (± 0.009) | 0.0305  (± 0.0024) | 2.773  (± 0.042) | 10.73 |

**Table S7 Taxonomic distributions of module hubs**

| name | zi | pi | taxa_roles | group | phylum | class | order | family | genus |
| --- | --- | --- | --- | --- | --- | --- | --- | --- | --- |
| Zotu37 | 2.73 | 0.23 | Module hubs | abundant | p:*Actinomycetota* | c: *Actinomycetota* | o: *Micrococcales* | f: *Micrococcaceae* | -- |
| Zotu117 | 2.83 | 0 | Module hubs | middle | p:*Pseudomonadota* | c: *Alphaproteobacteria* | o: *Sphingomonadales* | f: *Sphingomonadaceae* | g: *Sphingomonas* |
| Zotu78 | 3.76 | 0 | Module hubs | abundant | p:*Pseudomonadota* | c: *Alphaproteobacteria* | o: *Rhizobiales* | f: *Xanthobacteraceae* | g:*Bradyrhizobium* |
| Zotu88 | 3.76 | 0 | Module hubs | middle | p: *Chloroflexota* | c: *Gitt-GS-136* | o: *--* | f:*--* | g:*--* |
| Zotu404 | 2.73 | 0.077 | Module hubs | middle | p:*Pseudomonadota* | c:*Gammaproteobacteria* | o: *Burkholderiales* | f: *Oxalobacteraceae* | g: *Noviherbaspirillum* |
| Zotu196 | 2.83 | 0 | Module hubs | middle | p: *Nitrospirota* | c:*Nitrospiria* | o: *Nitrospirales* | f: *Nitrospiraceae* | g: *Nitrospira* |
